# Long-term vaccination strategies to mitigate the impact of SARS-CoV-2 transmission: A modelling study

**DOI:** 10.1371/journal.pmed.1004195

**Published:** 2023-11-28

**Authors:** Alexandra B. Hogan, Sean L. Wu, Jaspreet Toor, Daniela Olivera Mesa, Patrick Doohan, Oliver J. Watson, Peter Winskill, Giovanni Charles, Gregory Barnsley, Eleanor M. Riley, David S. Khoury, Neil M. Ferguson, Azra C. Ghani

**Affiliations:** 1 School of Population Health, Faculty of Medicine and Health, University of New South Wales, Sydney, Australia; 2 MRC Centre for Global Infectious Disease Analysis, Jameel Institute, School of Public Health, Imperial College London, London, United Kingdom; 3 Institute for Health Metrics and Evaluation, University of Washington, Seattle, United States of America; 4 London School of Hygiene and Tropical Medicine, London, United Kingdom; 5 Institute of Immunology and Infection Research, School of Biological Sciences, University of Edinburgh, Edinburgh, United Kingdom; 6 Kirby Institute, University of New South Wales, Sydney, Australia; Universitair Medisch Centrum Utrecht, NETHERLANDS

## Abstract

**Background:**

Vaccines have reduced severe disease and death from Coronavirus Disease 2019 (COVID-19). However, with evidence of waning efficacy coupled with continued evolution of the virus, health programmes need to evaluate the requirement for regular booster doses, considering their impact and cost-effectiveness in the face of ongoing transmission and substantial infection-induced immunity.

**Methods and findings:**

We developed a combined immunological-transmission model parameterised with data on transmissibility, severity, and vaccine effectiveness. We simulated Severe Acute Respiratory Syndrome Coronavirus 2 (SARS-CoV-2) transmission and vaccine rollout in characteristic global settings with different population age-structures, contact patterns, health system capacities, prior transmission, and vaccine uptake. We quantified the impact of future vaccine booster dose strategies with both ancestral and variant-adapted vaccine products, while considering the potential future emergence of new variants with modified transmission, immune escape, and severity properties. We found that regular boosting of the oldest age group (75+) is an efficient strategy, although large numbers of hospitalisations and deaths could be averted by extending vaccination to younger age groups. In countries with low vaccine coverage and high infection-derived immunity, boosting older at-risk groups was more effective than continuing primary vaccination into younger ages in our model. Our study is limited by uncertainty in key parameters, including the long-term durability of vaccine and infection-induced immunity as well as uncertainty in the future evolution of the virus.

**Conclusions:**

Our modelling suggests that regular boosting of the high-risk population remains an important tool to reduce morbidity and mortality from current and future SARS-CoV-2 variants. Our results suggest that focusing vaccination in the highest-risk cohorts will be the most efficient (and hence cost-effective) strategy to reduce morbidity and mortality.

## Abstract 2

In a modelling study, Alexandra Hogan and colleagues investigate long term vaccination strategies to mitigate the impact of SARS-CoV-2 transmission.

## Author summary

**Why was this study done?:**

- Severe Acute Respiratory Syndrome Coronavirus 2 (SARS-CoV-2), the virus causing Coronavirus Disease 2019 (COVID-19), is now endemic globally.
- Vaccination remains important to reduce hospitalisations and deaths.
- Health authorities need to consider how frequently booster doses will be required and in which age groups.
- They also need to know the additional value of switching to vaccines that have been adapted to match more recently circulating variants of concern.

**What did the researchers do and find?:**

- We developed a mathematical model that captures the continued circulation and evolution of the SARS-CoV-2 virus in the presence of widespread infection-induced immunity from past exposure as well as vaccine-induced immunity from primary vaccination campaigns in 2021/2022.
- We used the model to explore different strategies for continued vaccination including the age group targeted, the frequency of boosting, and whether the vaccine was adapted to match more recent variants.
- In both high-income and low-middle-income settings, regardless of whether there was a high level of transmission in 2020/21 or a zero-COVID policy, we found that the most cost-effective vaccination strategy was to boost those at highest risk.
- We found that the current variant-adapted vaccines could avert nearly twice as many hospitalisations and deaths compared to the ancestral vaccines, and that updating these vaccines each year—as is done for seasonal influenza—could avert a further 30% of hospitalisations and deaths.

**What do these findings mean?:**

- Continued booster vaccinations will remain important to reduce both hospitalisations and deaths but should be targeted towards the highest risk groups.
- The estimates provided here can help to inform discussions around value for money by comparing the cost-effectiveness of COVID-19 vaccination to other health programmes.
- The precise values are limited by uncertainty as to whether the virus will continue to evolve, whether any new variants may emerge, and the additional protection provided by further booster doses given the widespread exposure of the population to infection.

## Introduction

The rapid development and delivery of vaccines to protect against Severe Acute Respiratory Syndrome Coronavirus 2 (SARS-CoV-2) infection and Coronavirus Disease 2019 (COVID-19) dramatically altered the course of the pandemic, saving an estimated 19.8 million lives in the first year of vaccination alone [1]. However, the effectiveness of COVID-19 vaccines wanes, with considerable declines against infection but slower declines against severe disease and death [2–8]. Thus, it is likely that continued booster programmes will be needed to maintain high effectiveness against severe disease and death, particularly in those at highest risk of more severe outcomes [9]. In addition, vaccine booster programmes have been successful in partially restoring effectiveness against severe disease and death when levels of existing vaccine protection have been eroded by the emergence of new variants that have resulted in immunological escape [10–12].

Many countries are continuing to evaluate how best to schedule regular boosting to protect against ongoing endemic circulation of the virus as well as against future epidemic waves with new variants. This is reflected in the World Health Organization (WHO) roadmap which, in the most recent update (March 2023), recommended different vaccination schedules for high, medium, and low priority use groups, with boosting restricted to the highest risk group (older adults, younger adults with significant comorbidities or severe obesity, adults with moderate to severe immunocompromising conditions, pregnant people, and frontline health workers) [9]. The benefit of such strategies in any given population will depend on the current stage of the vaccine programme, including the supply of vaccine doses, and the extent to which these doses are matched to the current circulating strains. It will also depend on the extent of infection-acquired immunity and the additional protection that this provides. In general, both cohort and test negative case-control studies have suggested an additional impact of past infection in addition to vaccine-induced immunity in providing protection against severe outcomes, although these studies were not designed to control for the timing of exposure versus vaccination [13–15]. However, in a population-based cohort with frequent access to COVID-19 testing that enabled the timing of exposures to be included in the analysis, hybrid immunity was demonstrated to provide higher levels of neutralising antibodies over time [16]. Lastly, the benefits of booster vaccination will depend on the extent to which any future variant replaces the current Omicron variant and whether it further evades existing immunity.

Throughout the COVID-19 pandemic, mathematical and computational modelling has been a key component of longer-term planning and has been widely used to inform decisions on future vaccine strategies [17]. A number of studies have focussed on either country-specific projections of epidemic progression and vaccine impact, or on allocation or prioritisation of the limited supply of doses (particularly in the early stages of vaccine rollout) [18–22]. More recently, models have been developed to consider longer-term strategies for continued vaccination. Data-driven approaches have used the relationship between neutralising antibody titres and protection to estimate individual-level protection [23–25], while transmission models have incorporated profiles for vaccine-induced and infection-induced immunity over time to estimate the direct and indirect impact of different vaccination and dosing strategies [26–28]. Planning for these scenarios, particularly considering potential future variant characteristics, has been identified as a global public health priority [29,30].

The aim of our study was to estimate the infections, hospitalisations, and deaths averted at the population level of a range of future COVID-19 vaccination strategies compared to a baseline of no further boosting. In contrast to other transmission modelling studies, we developed a transmission model that explicitly incorporates hybrid immunity (immunity induced by both infection and vaccines), in order to capture the interactions between past exposure and vaccination. We did this by embedding an existing within-host model of underlying immunity dynamics and protection against infection and severe disease (similar to that presented in Khoury and colleagues [24]), which has been previously fitted to vaccine effectiveness data from England [31] within a population-based virus transmission model for SARS-CoV-2. We used this model to explore the impact of different targeted booster strategies—evaluating age-based targeting, different frequencies of boosting, and the value of using variant-adapted vaccines (both the 2022 bivalent products and theoretical yearly updated vaccines)—under the assumption that the Omicron variant continues to gradually evolve. We additionally consider the potential impact of the emergence of a new variant and the likelihood that vaccination could sufficiently mitigate its impact.

## Methods

### Immunological model

The immunological model is as described in Hogan and colleagues [31]. Briefly, we followed the approach in Khoury and colleagues [25], in which neutralising antibody titre is assumed to be a correlate of protection against SARS-CoV-2 infection, severe disease, and death over time. Such a model does not necessarily exclude other immune mechanisms playing a role in protection—including T cell-mediated immunity—but rather makes the underlying assumption that the patterns of protection over time can be related to the trends observed in the neutralising antibody titre. We therefore define these underlying dynamics of immunity as an individual’s immunity level (IL) [31]. We further assume that an individual’s IL decays according to a biphasic exponential decay function, where an initial faster period of decay is followed by a longer period of slow decay [24,25]. We then assume logistic relationships between IL and effectiveness to capture time-varying vaccine protection against mild disease (infection) and hospitalisation over time, with the logistic function parameterisation capturing higher protection against severe outcomes [25]. We use model parameters against the Delta and Omicron variants for 2 vaccine products—the Oxford/AstraZeneca AZD1222 vaccine and the Moderna mRNA-1273 vaccine (Table A in S1 Text). The resulting pattern of IL and vaccine effectiveness over time are shown in Hogan and colleagues [31]. To capture loss of immune recognition against Omicron and future variants, we estimate a multiplicative scaling factor (referred to as the variant fold reduction, VFR) to reflect the reduced neutralization of a given variant for each modelled vaccine. This VFR for the ancestral vaccines is based on our estimates of the degree of immune escape obtained by fitting the immunological model to vaccine effectiveness data against the Delta and Omicron (BA.1/2) variants [31].

We use the same approach to capture infection-induced immunity and its interaction with vaccine-induced immunity, where each infection is assumed to generate a boost to IL of 1, i.e., equivalent to that measured in convalescents [25]. In our model, this corresponds to a mean protection against reinfection of 80% over 180 days, similar to estimates obtained in a recent study of infection-induced protection against Omicron BA.4 and BA.5 subvariants which found 76.2% [95% CI 66.4%, 83.1%] protection against symptomatic reinfection [32]. This level of boost is higher than that observed under primary vaccination, potentially representing a broader and longer-lasting immune response and resulting in higher infection-induced protection. We additionally performed sensitivity analyses to this assumption exploring boosts to IL of 0.75 and 1.25, corresponding to protection against reinfection over 180 days of 65% and 89%, respectively. In the absence of definitive data to suggest otherwise [33], the infection-induced IL is assumed to decay at the same rate as following booster vaccination. We include a sensitivity analysis to this assumption by reducing the rate at which immunity is lost by either 75% or 50% (i.e., increasing the durability of protection). Each infection or vaccine dose results in an additive increase in IL, with an upper limit on the total level of vaccine- or infection-induced IL for each individual [34].

We assume that following the emergence of a variant, the infection-induced IL developed through exposure to previous variants is reduced by the VFR in the same way that vaccine-induced protection is reduced. However, subsequent infections with the new variant are assumed to match and therefore are not reduced by the VFR. The degree of matched protection is set to capture strain-specific protection against infection without explicitly modelling each variant (see S1 Text).

We capture the additional benefit of variant-adapted vaccines by increasing the immunogenicity generated upon vaccination (so as to reduce the impact of the VFR once variant-adapted vaccines are introduced). Khoury and colleagues estimated a 1.61-fold [95% CI 1.5, 1.8] relative neutralization titre for variant-adapted compared to ancestral vaccines based on the vaccines available at the end of 2022 [24]. To capture the initial variant-adapted vaccines (i.e., the bivalent vaccines against BA.1/2 and BA.4/5), we therefore apply an increase to the vaccine-induced IL from dose 4 onwards. This partially counteracts the impact of immune escape (the VFR) of the circulating SARS-CoV-2 strain in 2022. This increase to the IL is calculated as the central estimate of the Omicron VFR divided by the relative neutralization titre of 1.61. In our first set of scenarios, we assume that this remains the primary vaccine and that no further updates are introduced. We refer to this schedule as “variant-adapted” vaccination.

We further explore the benefits of introducing a vaccine that is updated each year. To do so, we assume that the vaccine is matched to the variant that is circulating 1 year earlier. In the model, at the time point that a new dose is scheduled to be rolled out to the eligible population, we obtain the level of immune escape (i.e., the value of the VFR) at exactly 1 year earlier. We then multiply the vaccine-induced IL by this prior VFR to obtain an increase in the level of vaccine protection, and apply this process from dose 5 onwards, for the applicable scenarios. We refer to this schedule as “yearly updated” vaccination.

### Population model and vaccine allocation

**Fig 1:**
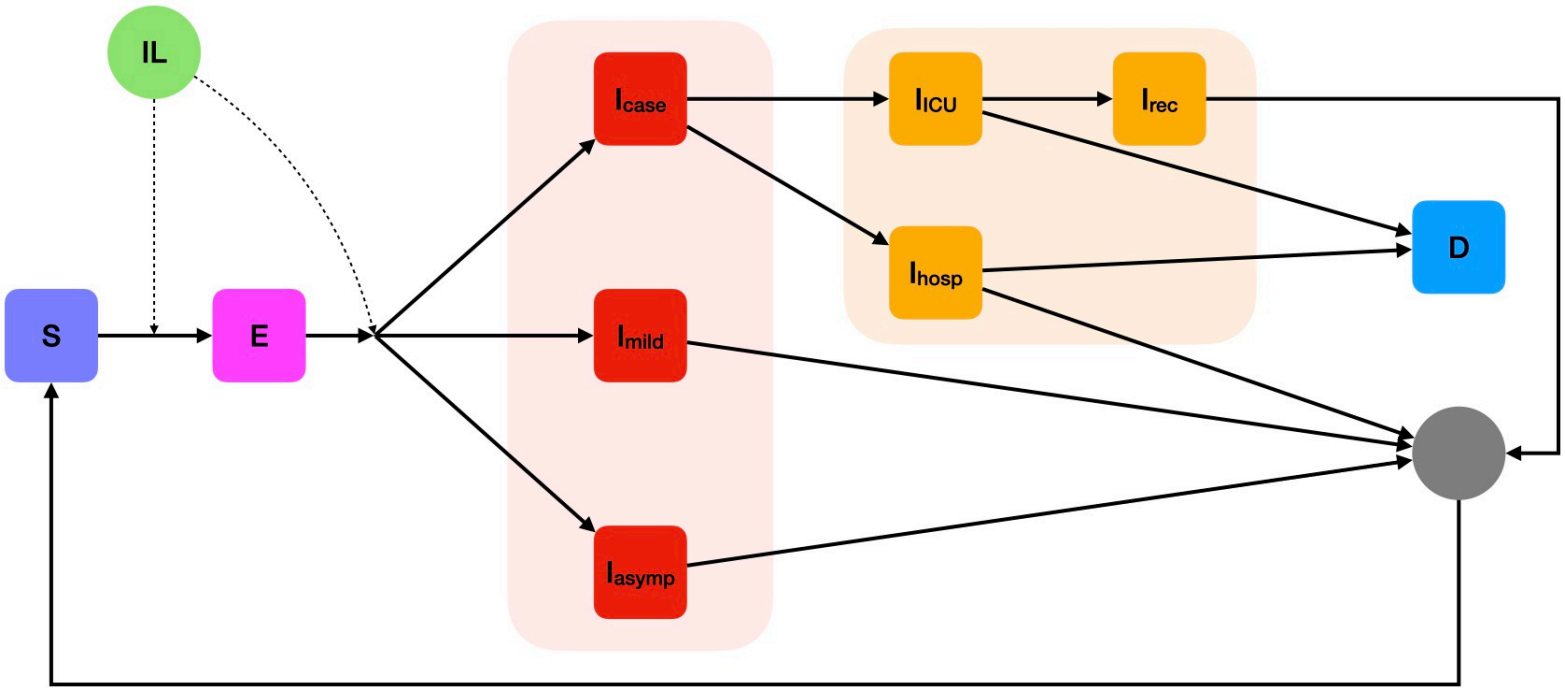
Schematic representation of the compartmental epidemiology of the stochastic individual-based model. The green circle denotes the IL, which increases in response to both exposure to infection and to vaccination and wanes over time (see section on the Immunological Model). The IL is tracked for each individual in the model and influences the probability of being infected (from susceptible S to exposed E) and of developing disease requiring hospitalisation (Icase) given a breakthrough infection. I_mild_ denotes mild symptomatic disease, I_asymp_ asymptomatic infection, I_hosp_ disease requiring hospitalisation (which is comprised of people requiring mechanical ventilation, people requiring oxygen, and people requiring neither), I_ICU_ disease requiring ICU admission, I_rec_ stepdown from ICU, and D death. ICU, intensive care unit; IL, immunity level.

To explore the population impact of vaccines, we developed a stochastic, individual-based model of SARS-CoV-2 transmission and vaccination (open-source at https://mrc-ide.github.io/safir/) [35]. The main transmission model captures the infection status of individuals as being in one of the follow states: susceptible; exposed, infectious with mild symptoms; infectious and asymptomatic; infectious requiring hospitalisation; hospitalised in a general ward; hospitalised in an intensive care unit (ICU); cases in recovery from ICU; and cases that have died (Fig 1). Within the model, each individual is assigned a 5-year age bin, with the bin sizes corresponding to the demographics of the population.

Vaccine-derived and infection-induced immune dynamics follow the immunological model described above. The model structure and epidemiology broadly mirror a previously published compartmental model, but these processes are instead implemented at the individual level [36]. This allows for immunity to be implemented at the individual level, capturing both vaccine- and infection-induced immunity, individual variation in this immunity, decay over time, and allowing for individual-level tracking of vaccine and infection history. Each individual’s level of immunity, or IL, can be boosted by infection or vaccination, and wanes over time according to the dynamics of the immunological model described above. This IL determines an individual’s risk of becoming infected and the subsequent risk of disease progression and hospitalisation. Transitions between epidemiological states are summarised in Fig 1 and Table B in S1 Text. The model was parameterised using published literature and was not explicitly fitted to data. Model parameters include those determining the natural history for SARS-CoV-2 infection (i.e., progression from infection to symptomatic and severe disease and infectivity), age-stratified probabilities of requiring hospital care, and the infection fatality ratio. These were obtained from Hogan and colleagues [36] and are reproduced in Table C in S1 Text. The model additionally captures differences between countries in demography, age-mixing patterns, and access to hospital facilities, using the parameterisation from Hogan and colleagues and Walker and colleagues [36,37].

The model structure also allows for a high degree of flexibility in dose and age-based vaccine prioritisation strategies. However, only 1 vaccine type can be modelled across the population; the results presented here are generated using the parameters from Hogan and colleagues [31] for the AZD1222 and mRNA-1273 vaccines. Vaccines are allocated according to an algorithm accounting for available stock, the age groups that are prioritised for each dose, minimum time delays between the receipt of subsequent doses, and coverage targets for each dose and age group following the approach taken in Hogan and colleagues [36]. This is described in further detail in S1 Text Section 1.5 and illustrated in Fig A in S1 Text.

### Settings and transmission

We consider 2 representative income settings—high-income countries (HICs) and lower-middle-income countries (LMICs)—and characterise each setting by contact patterns and demography [36,37]. In LMICs, we assume healthcare system capacity is limited; once modelled hospitalised cases exceed a threshold in these settings, infected individuals who require hospital care experience worse outcomes [37]. In HICs, we assume no limit to healthcare capacity due to surge provisions.

We further stratify the current epidemiological state of countries into 3 categories. “Category 1” represents countries that have experienced substantial past transmission (and hence have a substantial level of infection-induced immunity) alongside a high level of access to vaccines. Many high- and upper-middle-income countries fall into this category—including countries in North America, Central/South America, the Middle East, and Europe. We created a representative epidemic profile for such countries, with a first wave occurring between March and May 2020, a second wave during the northern hemisphere winter of 2020/21, and transmission gradually increasing (interventions being relaxed) from mid-2021 (Fig B in S1 Text). This broadly characterises a northern hemisphere setting but does not include seasonality. “Category 2” are countries that have experienced substantial prior transmission and have had limited distribution of vaccines. Many low- and lower-middle-income countries fall into this category (although we note that several LMICs have successfully limited transmission). For these countries, we model a similar background epidemic to that in Category 1 (Fig B in S1 Text) but with fewer interventions in place during 2021. “Category 3” countries are those that successfully interrupted transmission for a substantial time period (“zero-COVID” countries, mostly in east Asia and the Pacific) and therefore have more limited infection-induced immunity, alongside high vaccine uptake. For these, we assume a gradual lifting of restrictions (Fig B in S1 Text) from late 2021. These scenarios, alongside our transmission parameters, are informed by fits of a similar compartmental model to the global pandemic in 2020–2021 [1].

In all settings, we assume that the Omicron variant (BA.1 and BA.2 subtypes) gradually replaces Delta over 1 month from end-November 2021. This replacement impacts infection- and vaccine-induced immunity, transmissibility, and severity (see S1 Text). Following the emergence of the Omicron variant, we then assume that the virus continues to evolve or “drift” over time, with a new variant regularly replacing the dominant variant. This can be considered to represent the gradual drift that is now being observed with the Omicron subvariants. In our model, we implement this by increasing both the level of transmission and the VFR (relative to Delta) every 4 months, to represent small increases in transmissibility and gradual immune escape. Antigenic cartography studies have shown continued evolution with substantial jumps within the Omicron variant [38–40]. However, translating these antigenic maps into the degree of immune escape (and hence our VFR parameter) remains challenging given the widespread exposure to SARS-CoV-2 that has now occurred. We therefore explore a range of values for the degree of both transmission and immune escape. We use a central value of 5% for the main analysis and include a sensitivity of the model outcome to values between 0% and 20% in S1 Text.

### Vaccine dose strategies

The maximum population-level coverage for each income setting is based on the current WHO-reported coverage by income setting, which on 20 June 2023 reported 75% and 48% for the primary series and booster doses, respectively, in HIC settings, and 61% and 19%, respectively, in LMIC settings [41]. For our analysis, in HIC settings we assume total maximum population-level coverage of 80% and 53% for the primary series and booster doses, respectively. In LMIC settings, we assume a maximum coverage of 75% in all age groups >10 years that translates in our average demography to 56% for the primary series. This is slightly lower than the WHO reported statistic but is representative of age-based patterns in these settings. In LMIC settings, given that the rollout to date of booster doses has been limited to date, but is still underway, for our simulations we assume a maximum 60% uptake of booster doses relative to the primary series, equating to 39% population-level coverage. This is higher than the current WHO-reported level to reflect ongoing expansion of booster programmes in these settings [41]. In all settings, we prioritise the oldest individuals with vaccines delivered sequentially to consecutive 5-year age groups until the target age group is vaccinated, and we assume that uptake is highest in older age groups, with age-based uptake informed by various WHO and country-level sources [42–46]. We undertook an additional scenario as a sensitivity analysis where 70% population-level coverage of the primary series is achieved in LMIC settings, based on WHO policy targets [47]. As we assume that only individuals 10 years and older (10+) are eligible for vaccination, this total population-level coverage corresponds to higher uptake within targeted groups and zero coverage within ineligible groups; the within-age group uptake for each setting is shown in Table D in S1 Text.

Vaccine distribution strategies are implemented as follows. For Categories 1 and 3 (HICs), vaccines are administered at a constant rate of 5% of the population receiving 1 dose per week, starting 1 January 2021, assuming the mRNA-1273 vaccine for the primary 2-dose series and booster to those 10+. After the primary doses and first booster dose, we then either cease to administer any additional doses, or administer at the same pace, one of the following strategies with a variant-adapted vaccine: annual or 6-monthly booster doses to the 75+ population; annual or 6-monthly booster doses to the 60+ population; or annual booster doses to the 10+ population. We additionally consider the outcome if all doses from dose 4 onwards are the ancestral mRNA-1273 vaccine instead of a variant-adapted product.

For Category 2 (LMICs), vaccines are administered at a maximum constant rate of 2% of the population immunised per week starting 1 April 2021. We assume the first 2 doses are the AZD1222 vaccine, the first booster (third dose) is with the mRNA-1273 vaccine, and subsequent doses are either with a variant-adapted vaccine (default) or the ancestral mRNA-1273 vaccine. We assume the primary series and first booster is administered to those aged 10+ according to the levels of uptake in Table D in S1 Text. We then either cease to administer any additional doses, administer annual booster doses to the 60+ population at the same pace, boost the 40+ population annually, or boost the 10+ population annually.

For Category 2 (LMICs), we additionally model a separate scenario where we consider the relative impact of administering doses to vaccinate the younger working-age population with their primary series, versus diverting those doses to vaccinate the older population with a booster dose. We commence vaccination with the AZD1222 vaccine from April 2021, delivering the primary 2-dose series to the 40+ population. Once the target coverage is achieved, vaccination is paused until the delay between the second and booster doses (12 months) is complete. We then construct the following scenarios. For the first, we vaccinate the 40+ population with booster doses, beginning with the oldest (80+) age group. For the second, we take the same number of doses that would be required to give boosters to 40+ years, and instead deliver these doses to individuals younger than 40 years (2 doses per person). We construct these rollout scenarios such that the daily doses delivered is equivalent between scenarios. We compare these outputs with the scenario where no additional doses are delivered beyond 2 doses to the 40+ population.

**Fig 2:**
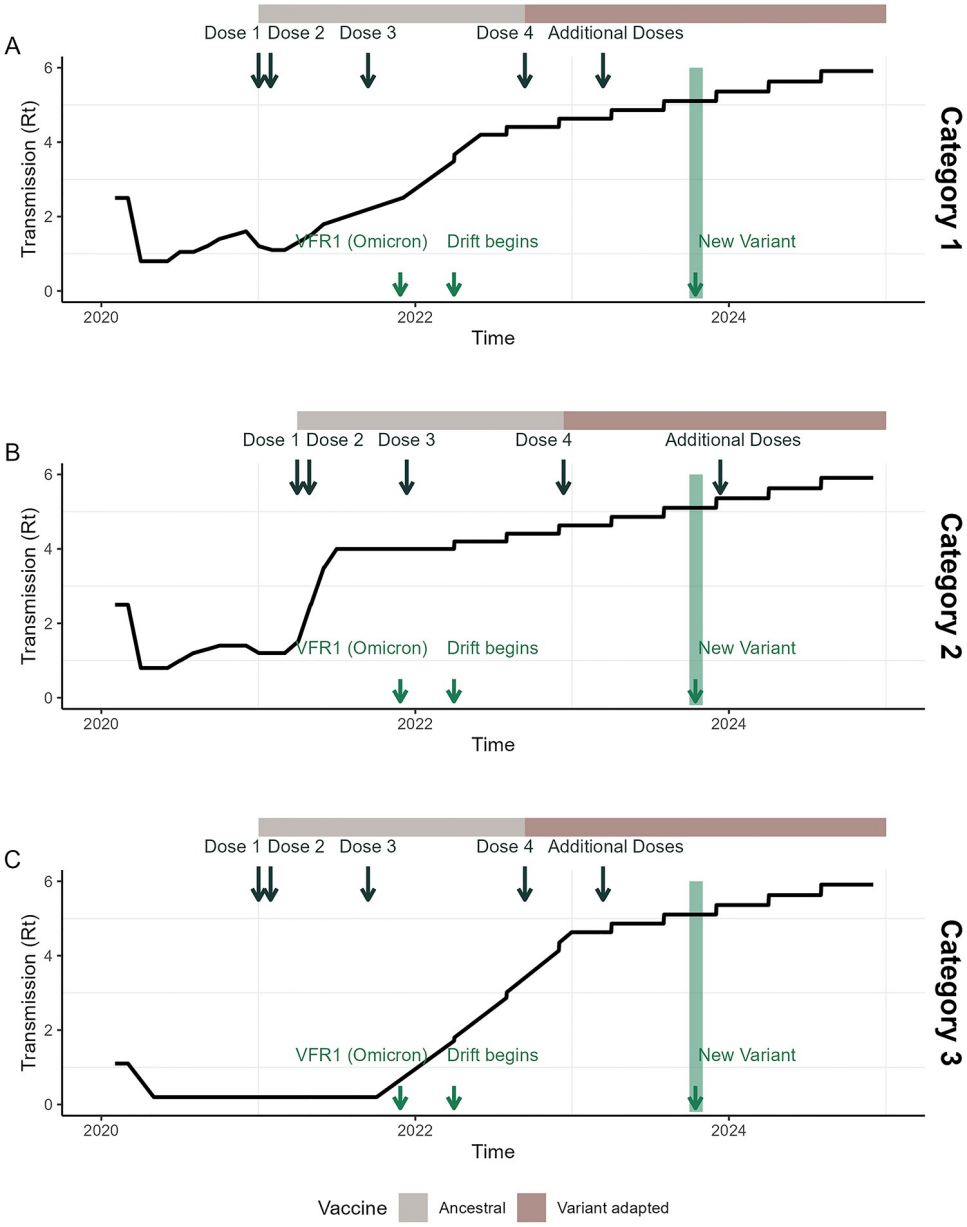
Summary of the scenarios explored. Key assumptions relating to the level of transmission, the timing of vaccine dose delivery, the vaccine product delivered, and the level of immune escape, are illustrated for each category. Category 1 represents high-income settings with historically high levels of SARS-CoV-2 transmission, Category 2 represents lower-middle-income settings with historically high levels of transmission, and Category 3 represents high-income settings with initially lower levels of transmission. Additional assumptions are summarised in Table D and E in S1 Text. SARS-CoV-2, Severe Acute Respiratory Syndrome Coronavirus 2; VFR, variant fold reduction.

Setting characteristics and vaccine rollout assumptions are summarised in Table E in S1 Text. The scenarios in each of the 3 settings are illustrated in Fig 2.

### Variant scenarios

In addition to our baseline scenario in which we mimic the continued gradual antigenic drift within the Omicron clade that is captured in the main scenarios, we explored the impact of the emergence of a more antigenically distant dominant variant. This was chosen to reflect a “worst-case scenario” given the ongoing uncertainty in the future evolution of the virus. We assume that this new variant emerges and replaces the Omicron variant between 1 and 31 October 2023 and that thereafter this new variant continues to gradually drift (as for the Omicron variant) for the remainder of the simulation time period (i.e., to end-2024). We consider 3 possible new variants with the following characteristics: (1) severity increased to that of Delta (“increased severity”); (2) VFR relative to Delta increased to 10 (“additional immune escape”) and hence an additional 2-fold reduction relative to Omicron (an antigenic shift away from Omicron of a similar magnitude to the shift from Delta to Omicron); and (3) both severity increased to Delta and VFR increased to 10 (“increased severity and immune escape”). These 3 scenarios represent a range of plausible scenarios (since they are based on what has been observed with previous variants) and also generate a reasonable worst-case scenario.

### Forward simulations

For each scenario, we repeat the model simulation across 50 random seeds, with a simulation population size of 1 million, and summarise the outputs by the median over these stochastic realisations. As the runs are based on a single model, we were unable to capture uncertainty in model structure. Furthermore, given the high degree of uncertainty in the future evolution of the virus and hence epidemic trajectories, we did not sample parameter uncertainty as these would give a sense of precision that is misleading. Instead, we evaluate the value of different vaccination strategies against a range of epidemic scenarios and address parameter uncertainty through sensitivity analyses. This is in line with the longer-term scenario methods used to support policy in situations of high uncertainty [48].

We use these outputs to calculate the daily infections and hospitalisations from 1 February 2020 to 31 December 2024, as well as the total infections, hospitalisations, and deaths from 1 July 2022 to the end of the simulation window. Given the high degree of uncertainty in the future course of the pandemic, these numerical values should be interpreted as indicative guides rather than predicted estimates.

### Impact and cost-effectiveness

The model outputs include infections, hospitalisations, and deaths over time alongside the number of vaccine doses that have been delivered. These outputs are used to estimate the number of vaccine doses required to avert X hospitalisations and deaths (where X is specified). We further estimate the incremental cost per hospitalisation and death averted of the vaccine strategies by calculating the total number of additional vaccine doses delivered, relative to the “3 doses only” (HIC) or “no additional doses” (LMIC) scenarios. These values are divided by the additional hospitalisations or deaths averted compared to the baseline scenario and multiplied by the assumed cost of each dose to obtain the incremental cost-effectiveness. We use 3 illustrative costs for the vaccine unit price (US $2, $20, or $50 per dose) as this price is known to vary between countries and it not available in the public domain. While vaccine prices may differ between products, as this information is not publicly available, we assume no difference in price between the different vaccine types [49]. These illustrative unit costs per dose are assumed to incorporate delivery and wastage.

## Results

**Fig 3:**
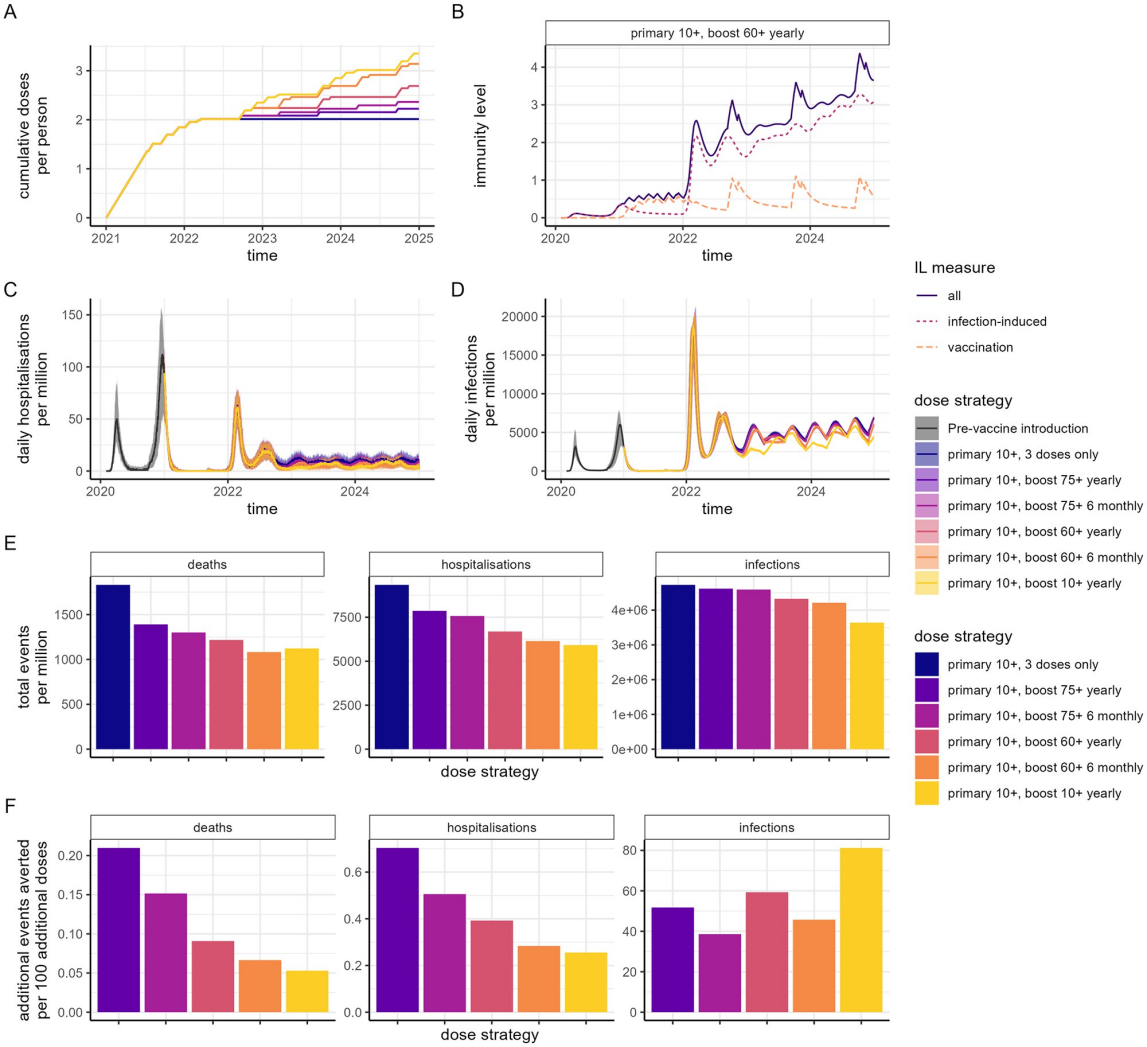
Impact of vaccination in an HIC setting with substantial prior transmission and high vaccine access (Category 1). We assume mRNA-1273 is implemented for the first 2 doses and the first booster (dose 3), and a variant-adapted vaccine for subsequent booster doses with no additional changes to the vaccine product (i.e., no further updating). (A) Cumulative doses delivered per person over time, for a range of dose delivery strategies. In all strategies, the primary series was delivered to individuals 10 years and older, with scenarios of no additional doses; annual or 6-monthly boosters to the 75+ years population; annual or 6-monthly boosters to the 60+ years population; or annual boosters to the 10+ years population. (B) Mean infection-induced (pink dotted), vaccine-induced (orange dashed), and total (purple solid) IL over time for the “primary 10+, boost 60+ yearly” dose strategy. (C) Daily hospitalisations and (D) daily infections per million population for the 6 dose strategies, where the trajectory prior to vaccine introduction is shown in dark grey. (E) Total events (deaths, hospitalisations, and infection) per million population between 1 July 2022 and 31 December 2024 for each dose strategy. (F) Additional events averted per 100 additional doses over the same period relative to the “primary 10+, 3 doses only” dose strategy. Results for the scenario where no additional variant emergence occurs beyond Omicron (i.e., constant transmission and no additional immune escape) are shown in Fig D in S1 Text. HIC, high-income country; IL, immunity level.

**Fig 4:**
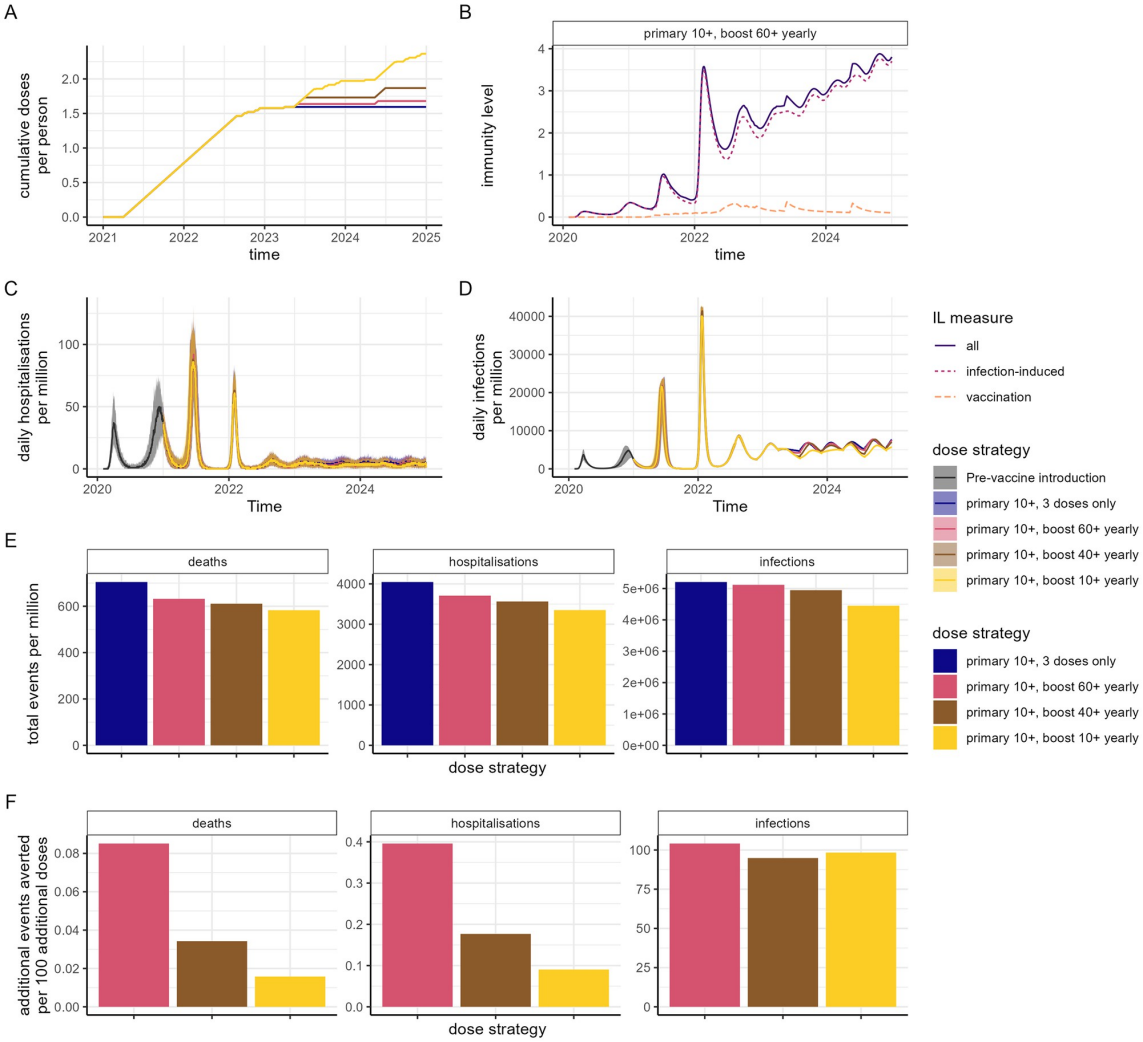
Impact of vaccination in an LMIC setting with substantial prior transmission and moderate vaccine access (Category 2). We assume AZD1222 is implemented for the first 2 doses, mRNA-1273 for the first booster (dose 3) and a variant-adapted vaccine for subsequent booster doses (doses 4 and 5) with no additional changes to the vaccine product (i.e., no further updating). (A) Cumulative doses delivered per person over time, for a range of dose delivery strategies. In all strategies, the primary series was delivered to individuals 10 years and older, with scenarios of no additional doses; annual boosters to the 60+ years population; annual boosters to the 40+ years population; or annual boosters to the 10+ years population. (B) Mean infection-induced (pink dotted), vaccine-induced (orange dashed), and total (purple solid) IL over time for the “primary 10+, boost 60+ yearly” dose strategy. (C) Daily hospitalisations and (D) daily infections per million population for the dose strategies, where the trajectory prior to vaccine introduction is shown in dark grey. (E) Total events (deaths, hospitalisations, and infection) per million population between 1 July 2022 and 31 December 2024 for each dose strategy. (F) Additional events averted per 100 additional doses over the same time period relative to the “primary 10+, 3 doses only” dose strategy. Results for the scenario where no additional variant emergence occurs beyond Omicron (i.e., constant transmission and no additional immune escape) are shown in Fig E in S1 Text. IL, immunity level; LMIC, lower-middle-income country.

Across all scenarios, provided Omicron remains the dominant variant, our model simulations show a pattern of low-level continued circulation of SARS-CoV-2 from early-2023 onwards (Figs 3, 4, and Fig D, E, and F in S1 Text). The precise levels of endemicity are sensitive to our assumptions regarding the evolution of SARS-CoV-2 represented in the model by the level of drift (Fig L in S1 Text) with our baseline assumption of 5% replicating the steady waves of infections and hospitalisations that have been observed in 2022/2023. Within the HIC settings modelled, we project fewer hospitalisations and deaths from mid-2022 in high transmission countries with prior transmission compared to those with minimal prior transmission (Fig 3C–3E and Fig F in S1 Text) due to the higher population immunity driven by hybrid immunity (Fig 3B and Fig F in S1 Text). In both settings, hospitalisations and deaths can be substantially reduced by continuing regular boosting at either 6- or 12-monthly intervals to the highest risk age groups but these strategies have less impact on infection incidence (Fig 3E and Fig F in S1 Text). In Category 1 countries, with an illustrative unit cost of $20 per dose for a variant-adapted vaccine, for yearly boosting this translates to $2,800 and $5,000 per hospitalisation averted for boosting 75+ and 60+ populations, respectively, and $9,500 and $22,000 per death averted (Table 1, values rounded to nearest $100). These same strategies are slightly more cost-effective in Category 3 countries due to the reduced alternative protection from prior infection-induced immunity. If the whole population (10+ years) is regularly boosted, we predict a substantial impact on transmission despite incomplete vaccine protection against infection, resulting in a more pronounced wave-like endemicity driven by population-level immune boosting and decay, and a lower endemic level (Fig 3C and 3D and Fig F in S1 Text). However, if the aim is solely to protect against hospitalisation and death, then we estimate that higher efficiency (events averted per vaccine dose) can be achieved by regular vaccination of those aged 60+ (Fig 3F and Fig F in S1 Text) with this reflected in the higher cost per hospitalisation or death averted of 10+ boosting (Table 1). The endemic prevalence—and hence the precise cost-effectiveness—is sensitive to our assumptions regarding the level of protection afforded by prior infection (Fig M in S1 Text) and the durability of infection-induced protection (Fig N in S1 Text). Higher assumed levels of protective immunity from past infection result in lower endemic prevalence and therefore lower cost-effectiveness of vaccination.

**Table 1:** Total additional infections, hospitalisations, and deaths averted, and total additional vaccine doses delivered for the Category 1 and 3 settings. We assume the mRNA-1273 vaccine is implemented for the first 2 doses and the first booster (dose 3), and a variant-adapted vaccine for subsequent booster doses with no additional changes to the vaccine product (i.e., no further updating). Impact is expressed relative to the scenario where the primary series plus a booster is delivered to the 10+ years population, with no additional doses. Totals are shown for the period from 1 July 2022 to 31 December 2024. Unless otherwise specified, we assume no additional variant emergence beyond Omicron and its subtypes. The “new variant worse-case scenario” refers to a scenario where a new variant replaces Omicron over 1 month, starting 1 October 2023, with VFR = 10 relative to Delta and severity similar to Delta. Values are the median estimate across 50 model simulations for each scenario. Total modelled events for each scenario are in S1 Table S6.

Vaccination scenario	Doses delivered per million population	Infections averted per thousand population	Hospitalisations averted per million population	Deaths averted per million population	Doses to avert one hospitalisation	Doses to avert one death	Cost per hospitalisation averted ($)			Cost per death averted ($)		
Unit cost per vaccine dose (illustrative)							2	20	50	2	20	50
HIC with substantial prior transmission and high existing vaccine coverage												
Boost 75+ yearly	210,015	109	1,477	441	142	476	284	2,844	7,110	952	9,524	23,811
Boost 75+ 6-monthly	350,025	135	1,770	531	198	659	396	3,955	9,888	1,318	13,184	32,959
Boost 60+ yearly	67,5570	401	2,651	614	255	1,100	510	5,097	12,742	2,201	22,006	55,014
Boost 60+ 6-monthly	112,5950	515	3,195	749	352	1,503	705	7,048	17,621	3,007	30,065	75,164
Boost 10+ yearly	133,7184	1,086	3,421	709	391	1,886	782	7,818	19,544	3,772	3,7720	94,301
Boost 60+ yearly, new variant worst-case scenario	675,570	2,511	13,206	3,851	51	175	102	1,023	2,558	351	3,509	8,771
Boost 10+ yearly, new variant worst-case scenario	1,337,184	3,046	14,451	4,103	93	326	185	1,851	4,627	652	6,518	16,295
HIC with limited prior transmission and high existing vaccine coverage												
Boost 75+ yearly	210,015	72	1,656	525	127	400	254	2,536	6,341	800	8,001	20,001
Boost 75+ 6-monthly	350,025	177	2,173	650	161	538	322	3,222	8,054	1,077	10,770	26,925
Boost 60+ yearly	675,570	390	3,105	732	218	923	435	4,351	10,879	1,846	18,458	46,145
Boost 60+ 6-monthly	1,125,950	459	3,687	862	305	1,306	611	6,108	15,269	2,612	26,124	65,310
Boost 10+ yearly	1,337,184	1,086	3,991	848	335	1,577	670	6,701	16,752	3,154	31,537	78,843
Boost 60+ yearly, new variant worst-case scenario	675,570	412	6,267	1,918	108	352	216	2,156	5,390	704	7,045	17,611
Boost 10+ yearly, new variant worst-case scenario	1,337,184	877	7,194	2,112	186	633	372	3,717	9,294	1,266	12,663	31,657

HIC, high-income country; VFR, variant fold reduction.

In LMIC populations that have experienced substantial prior transmission and have low vaccine coverage, we project a similar pattern of endemic prevalence to HIC that have experienced substantial prior transmission due to the high levels of infection-induced immunity (Fig 4). However, hospitalisations and deaths are projected to be lower in LMIC compared to HIC settings due to a younger population combined with the broader protection generated from infection-induced immunity compared to vaccine-induced immunity. Hence, the costs per hospitalisation and death averted for future vaccination strategies are substantially higher than in HICs at a given vaccine price (Table 2). At an illustrative unit cost of $2 per vaccine dose delivered (for a variant-adapted vaccine but based on costs in these settings [49]) boosting the 60+ population yearly would translate to $500 per hospitalisation averted and $2,400 per death averted. Total modelled doses, infections, hospitalisations, and deaths for each category and vaccination scenario are shown in Tables H and I in S1 Text, with the estimated impacts for the WHO vaccine coverage targets in LMIC settings shown in Fig H in S1 Text and Table J in S1 Text.

**Table 2:** Total additional infections, hospitalisations, and deaths averted, and total additional vaccine doses delivered for the Category 2 setting. We assume AZD1222 is implemented for the first 2 doses, mRNA-1273 for the first booster (dose 3), and a variant-adapted vaccine for subsequent booster doses (doses 4 and 5) with no additional changes to the vaccine product (i.e., no further updating). Impact is expressed relative to the scenario where the primary series plus a booster is delivered to the 10+ years population, with no additional doses. Totals are shown for the period from 1 July 2022 to 31 December 2024. Unless otherwise specified, we assume no additional variant emergence beyond Omicron and its subtypes. The “new variant worse-case scenario” refers to a scenario where a new variant replaces Omicron over 1 month, starting 1 October 2022, with VFR = 10 relative to Delta and severity similar to Delta. Values are the median estimate across 50 model simulations for each scenario. Total modelled events for each scenario are in S1 Table S8, with the total modelled events for the WHO coverage target scenario in S1 Table S10.

Vaccination scenario	Doses delivered per million population	Infections averted per thousand population	Hospitalisations averted per million population	Deaths averted per million population	Doses to avert 1 hospitalisation	Doses to avert one death	Cost per hospitalisation averted ($)			Cost per death averted ($)		
Unit cost per vaccine dose (illustrative)							2	20	50	2	20	50
LMIC with substantial prior transmission and low existing vaccine coverage: default coverage target assumption												
Boost 60+ yearly	85,128	89	337	72	253	1,182	505	5,052	12,630	2,365	23,647	59,117
Boost 40+ yearly	273,060	259	482	93	567	2,936	1,133	11,330	28,326	5,872	58,723	146,806
Boost 10+ yearly	769,254	756	695	121	1,107	6,357	2,214	22,137	55,342	12,715	127,149	317,874
Boost 60+ yearly, new variant worst-case scenario	85,128	71	505	222	169	383	337	3,371	8,429	767	7,669	19,173
Boost 10+ yearly, new variant worst-case scenario	769,254	568	984	329	782	2,338	1,564	15,635	39,088	4,676	46,763	116,908

LMIC, lower-middle-income country; VFR, variant fold reduction; WHO, World Health Organization.

We additionally find that in LMIC settings with high prior transmission, prioritising booster vaccinations in the highest-risk population has a slightly greater public health impact, reducing hospitalisations and deaths by approximately 5% to 10%, compared to using these same doses to immunise younger age groups in an effort to reduce transmission (Fig G in S1 Text and Table M in S1 Text). We observed a larger difference in impact of these strategies for hospitalisations and deaths compared to infections (Fig G in S1 Text).

**Fig 5:**
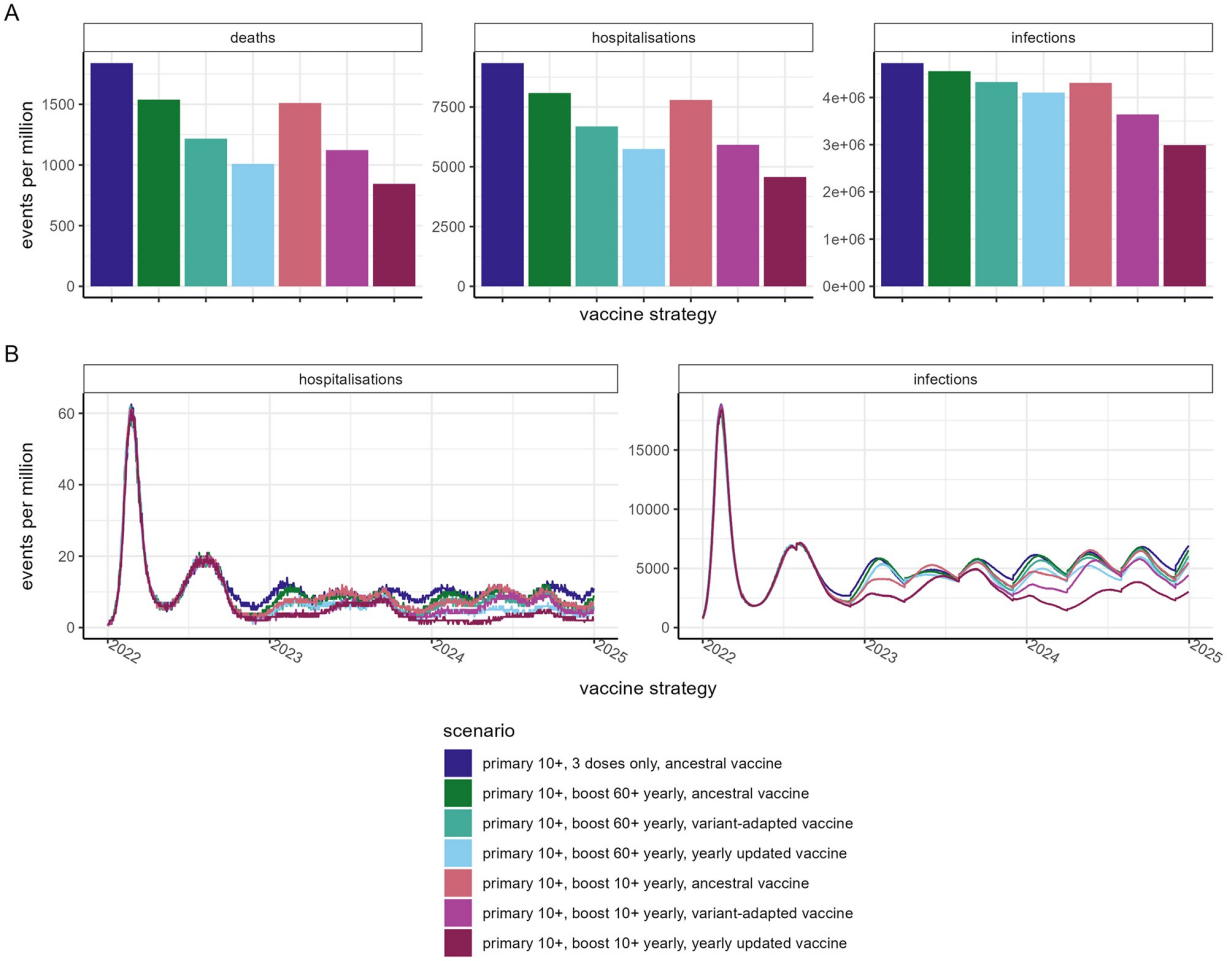
Comparison of vaccine impact for different ancestral and variant-adapted vaccine scenarios. **Results are shown for the HIC setting with substantial prior transmission (Category 1).** For all vaccine strategies, we assume mRNA-1273 is implemented for the first 2 doses and the first booster (dose 3). Following this, either no additional doses are administered, the ancestral vaccine is administered for subsequent doses, a variant-adapted vaccine is administered from dose 4, with no additional changes to the vaccine product (“variant-adapted vaccine”), or a variant-adapted vaccine is administered for dose 4, and subsequent doses are continually adapted based on the level of immune escape 12 months beforehand (“yearly updated vaccine”). (A) Summary events (deaths, hospitalisations, and infections) per million population for the different vaccine strategies between July 2022 and December 2024, and (B) daily hospitalisations and infections per million population. Values are reported in Table N in S1 Text. HIC, high-income country.

Fig 5 and Fig I and J in S1 Text compare the relative impact of variant-adapted vaccines and a yearly updated vaccine relative to continued administration of the ancestral vaccine as a booster. We estimate that switching to a variant-adapted vaccine product compared to continuing with the ancestral vaccine could avert around twice as many infections, hospitalisations, and deaths, and thus would reduce the cost per hospitalisation or death by around approximately 50% over the time frame considered, assuming the cost per vaccine dose is equivalent. Updating the vaccine each year to match the variant that was circulating 12 months previously (akin to seasonal influenza vaccination strategies) is estimated to avert approximately 50% more infections and around 30% more hospitalisations and deaths. The cost-effectiveness of such a strategy will depend on the additional costs associated with frequent vaccine updates.

**Fig 6:**
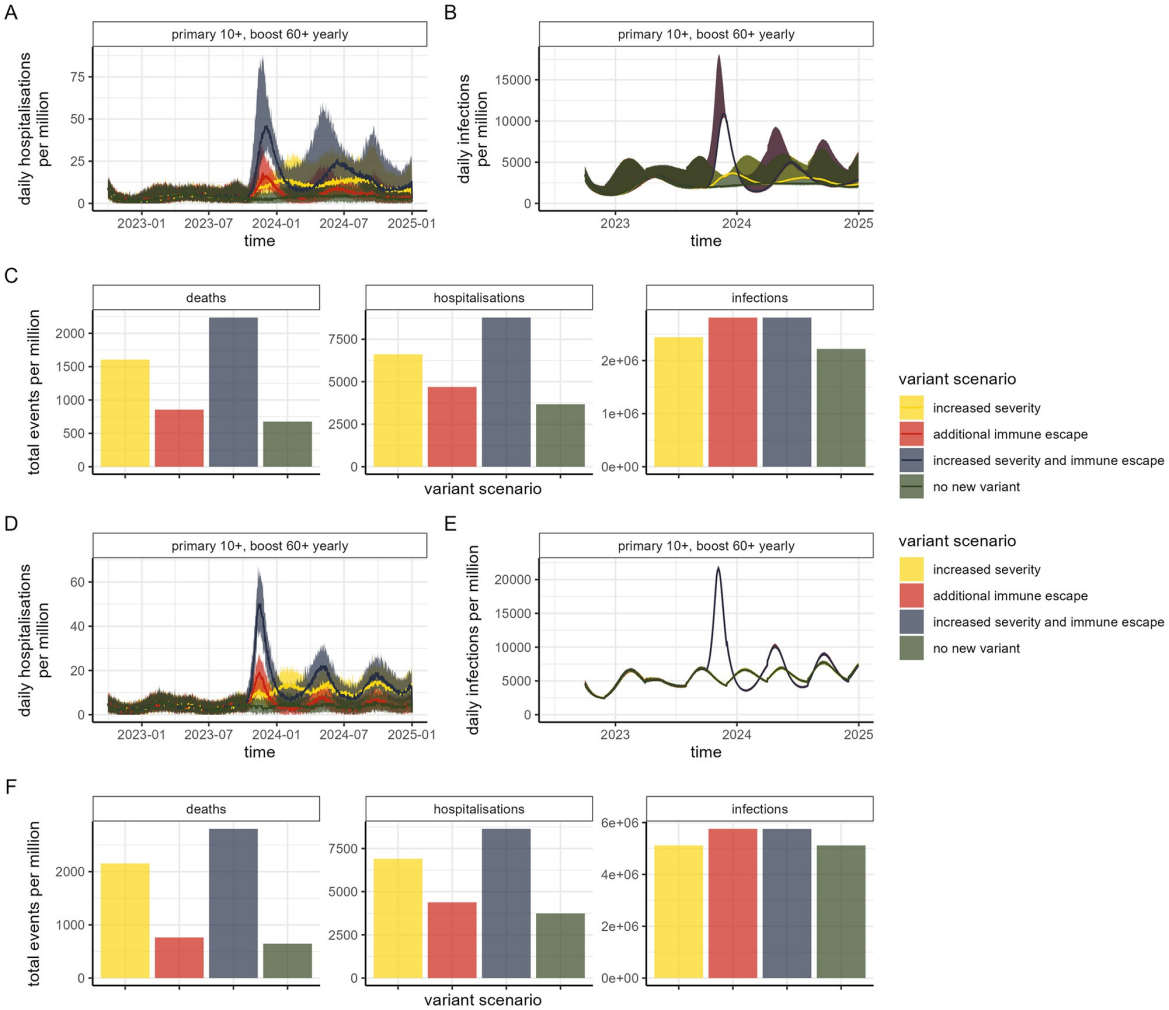
Impact of vaccination in future scenarios where an additional variant of concern emerges from 1 October 2023. We assume a variant-adapted vaccine is implemented from dose 4 with no additional changes to the vaccine product (i.e., no further updating). Three variant scenarios are shown: increased severity, where the risk of hospitalisations and severe disease reverts to that of Delta (yellow); additional immune escape, where the VFR increases to 10 (red); and increased severity and immune escape, which assumes both Delta severity and a VFR of 10 (blue). This is compared to the scenario with no new variant (green). (A) Daily hospitalisations and (B) daily infections per million population for the HIC setting with substantial prior transmission and high vaccine access (“Category 1”). (C) Total events (deaths, hospitalisations, and infections) per million population for each variant scenario for the “Category 1” setting, between 1 July 2022 and end-2024. (D) Daily hospitalisations and (E) daily infections per million population for the LMIC setting with substantial prior transmission and low vaccine access (“Category 2”). (F) Total events (deaths, hospitalisations, and infections) per million population for each variant scenario for the “Category 2” setting, between 1 July 2022 and end-2024. Results for the Category 3 setting are in Fig K in S1 Text. Values are reported in Table O in S1 Text. HIC, high-income country; LMIC, lower-middle-income country; VFR, variant fold reduction.

A completely new variant—simulated here as emerging in October 2023—that replaces the Omicron variant and its subtypes could rapidly result in a new epidemic wave, with the magnitude of this wave dependent on the properties of the variant (Fig 6A, 6B, 6D and 6E). Under a plausible worst-case scenario in which the variant has a similar severity profile to Delta and exhibits a shift in antigenic space similar to Omicron and therefore twice as far from the vaccines as was observed for Omicron, we predict levels of demand on health services similar to or exceeding those experienced during 2020 (Fig 6C and 6F). Under such a scenario, continued boosting scenarios become substantially more cost-effective despite the lower overall effectiveness of vaccination (Tables 1 and 2).

Our results are sensitive to the assumed future level of virus drift (Fig L in S1 Text). In our main analysis, we assume a level of 5% every 4 months, but additionally illustrate the future impact of vaccination in the absence of any continued evolution of the virus beyond the emergence of the Omicron variant (Fig D and E in S1 Text). A lower level of assumed drift results in a lower health impact of COVID-19 (Table G and I in S1 Text), and therefore lowers the cost-effectiveness of vaccination (Table K and L in S1 Text).

## Discussion

Assuming continued circulation of the Omicron variant with a degree of virus drift, our simulations mimic continuation of the pattern of low levels of SARS-CoV-2 circulation from late 2022 onwards, with waves of infection, hospitalisation, and deaths driven by the continued evolution of the virus. Our results indicate that the greatest impact on this endemic prevalence can be achieved through regular boosting of the 10+ years population. However, the efficiency and cost-effectiveness of a boosting programme depends on the outcome measure; a strategy targeting only 75+ years averts the largest number of deaths and hospitalisations per dose, whereas a strategy targeting 10+ has the largest reduction in infections but is relatively inefficient in reducing severe outcomes. Similar patterns were obtained regardless of whether the country has previously experienced large waves of infection (and therefore has considerable infection-induced immunity) or whether the country had previously pursued a zero-COVID policy. However, in the zero-COVID policy setting we generally estimate higher numbers of hospitalisations and deaths compared to settings with both high prior transmission and vaccine coverage. The health burden is higher because vaccine-induced immunity alone is estimated to be less protective than the combination of vaccine- and infection-induced immunity (or hybrid immunity) [33,50,51].

Cost-effectiveness will likely be the metric driving future vaccine strategies. Our results suggest that across all settings considered, targeting the highest risk group is likely to be the most cost-effective strategy as judged by the cost of preventing either a hospitalisation or a death. With variant-adapted vaccines now routine in high-income settings and also being used in some lower-income settings, we estimate that switching to such a variant-adapted vaccine could reduce the cost of preventing a hospitalisation or a death by around half. Switching to a yearly updated variant-adapted vaccine—as indicated by recently released United States Food and Drug Administration (FDA) guidance for manufacturers [52]—is projected to increase the cost-effectiveness by approximately 30%.

It should be noted that our estimates of variant-adapted vaccine effectiveness are based on immunogenicity studies and will therefore be sensitive to our fitted relationship between the underlying immunological mechanism and protection. The results will also be sensitive to our assumed continued level of antigenic drift. Furthermore, to capture the full cost-effectiveness further information is needed on variant-adapted and yearly updated vaccine effectiveness and the comparative unit price of new products. Importantly, we found that even continuing administration of the ancestral vaccine products (or existing variant-adapted vaccines where they have been introduced) is likely to reduce infections and severe outcomes in all settings. Furthermore, while estimating cost-effectiveness based on reductions in hospitalisations and deaths is relatively straightforward, such analyses do not account for the impact of high infection levels on long COVID incidence. COVID-19 hospitalisations and deaths in HICs have been concentrated in elderly populations; in contrast, long COVID is reported across a wider age range [53,54]. Comprehensive cost-effectiveness analyses therefore need to consider the potential longer-term effects of this illness on quality of life and future productivity.

Our analysis is caveated by the uncertainty in the timing and impact of any new variant. By definition, any variant that can replace the currently circulating Omicron variant will either need to be more transmissible or exhibit significant immune escape. Given that antigenic mapping studies suggest that, to date, there is no clear pattern of antigenic drift [38–40], our assumptions should be regarded as plausible but illustrative rather than predictive. Furthermore, translating the antigenic cartography into its associated impact on transmissibility and/or immune escape remains difficult and further research is needed to better quantify this relationship. In addition, there is concern that a new variant could exhibit the increased severity seen with Delta. Our results illustrate that, under a worst-case scenario, an epidemic wave of similar magnitude to those experienced in the first year of the pandemic could occur, even with regular boosting to the highest risk age groups using variant-adapted vaccines. This ongoing uncertainty provides a further challenge in valuing vaccination programmes; while widespread boosting could mitigate the impact of a new variant and would be substantially more cost-effective if it did arise, such a boosting strategy is inefficient and therefore unlikely to be cost-effective if such a variant does not emerge. It will therefore be important for countries to consider other mitigation strategies such as timely provision of antivirals.

Our study has several limitations. First, the timing and magnitude of waves of SARS-CoV-2, the dominant circulating variant during these waves (particularly over the past 12 months), the timing and stringency of non-pharmaceutical interventions, and the vaccination response, has varied widely between countries. Our results are therefore illustrative and more detailed country-specific modelling will likely be required. Second, our immunological model is necessarily a simplification of the complex underlying immune response. The quality and durability of this response will likely vary by age; however, there are currently insufficient data to explore the impact of age on waning efficacy or immune escape from booster doses due to the shorter follow-up in younger populations. Furthermore, the degree to which prior immunity protects against future variants (including the currently circulating Omicron subtypes that are antigenically distinct from BA.1 estimates in the data used here [38,40]) remains uncertain. The durability of infection-induced immunity compared to vaccine-induced immunity remains uncertain; while studies using antibody data as a surrogate of protection have suggested that vaccine-induced immunity can be more durable [23,55], there is a growing body of evidence demonstrating the importance of cell-mediated immunity in providing longer-term protection for both vaccine-induced and infection-induced immunity [56–58]. It has been suggested that immune imprinting could reduce the effectiveness of continued boosting across different populations [59]. This effect was not included in our model and would lower the public health impact and cost-effectiveness of the vaccination strategies that we considered. Finally, we only provide illustrative costing metrics as a first step towards broader cost-effectiveness analyses. Such analyses will depend on longer-term follow-up of the quality of life and persistence of disability following both mild infections and hospitalisation.

Our analyses illustrate the importance of continued booster doses as part of the wider public health response to ongoing endemic transmission of SARS-CoV-2 [23,60]. Prioritising boosters to high-risk and older populations is an efficient strategy in terms of reducing hospitalisations and death, while managing finite healthcare resources, but further data are required to understand the cost-effectiveness of vaccinating a wider age group to protect against the consequences of long COVID.

## Supporting information

S1 Text Supplementary details including further mathematical description of the model and parameters. Fig A. Schematic diagram illustrating the COVID-19 vaccine allocation algorithm. Fig B. Modelled trajectories of the reproduction number R_t_ over time. Fig C. Exemplar demographic patterns for each of the 2 income settings. Fig D. Impact of vaccination in a high-income country setting with substantial prior transmission and high vaccine access, assuming no additional variant emergence beyond Omicron (i.e., constant transmission and no additional immune escape, or no “drift”). Fig E. Impact of vaccination in a lower-middle-income country setting with substantial prior transmission and moderate vaccine access, assuming no additional variant emergence beyond Omicron (i.e., constant transmission and no additional immune escape, or no “drift”). Fig F. Impact of vaccination in a high-income country setting with minimal prior transmission and high vaccine access (Category 3). We assume mRNA-1273 is implemented for the first 2 doses and the first booster (dose 3) and a variant-adapted vaccine for subsequent booster doses. Fig G. Impact of vaccination in a low-middle-income country setting with substantial prior transmission and low vaccine access, where individuals 40+ years are initially targeted. Fig H. Impact of vaccination in a lower-middle-income country setting with substantial prior transmission and moderate vaccine access (Category 2), assuming WHO coverage targets. Fig I. Comparison of vaccine impact for different ancestral and variant-adapted vaccine scenarios for the lower-middle-income country setting with substantial prior transmission (Category 2). Fig J. Comparison of vaccine impact for different ancestral and variant-adapted vaccine scenarios for the high-income country setting with minimal prior transmission (Category 3). Fig K. Impact of vaccination in future scenarios where an additional variant of concern emerges from 1 October 2022, in a high-income setting with minimal prior transmission and high vaccine access (Category 3). We assume a variant-adapted vaccine is implemented from dose 4. Fig L. Sensitivity of the model output to the level of “drift” in a high-income setting with substantial prior transmission (Category 1). Fig M. Sensitivity of model results to assumptions regarding the level of protection afforded by infection in a high-income setting with substantial prior transmission (Category 1). Fig N. Sensitivity of model results to the decay rate of protection following recovery from SARS-CoV-2 infection in a high-income setting with substantial prior transmission (Category 1). Table A. Prior and posterior parameter estimates for the immunological model. Table B. Transmission model state transitions. Table C. Transmission parameter description and values. Table D. Default scenarios and assumptions for vaccine uptake within targeted age groups for each of the 2 income settings. Table E. Default scenarios and assumptions for the 3 broad modelled categories of country and epidemiological state. Table F. Total doses delivered, infections, hospitalisations, and deaths for each of the Category 1 and 3 settings, for a range of vaccine dose strategies and variant scenarios. We assume mRNA-1273 is implemented for the first 2 doses and the first booster (dose 3), and a variant-adapted vaccine for subsequent booster doses. Table G. Total doses delivered, infections, hospitalisations, and deaths for each of the Category 1 and 3 settings, for a range of vaccine dose strategies and variant scenarios. We assume no additional variant emergence beyond Omicron (i.e., constant transmission and no additional immune escape, or no “drift”). Table H. Total doses delivered, infections, hospitalisations, and deaths for the Category 2 setting, for a range of vaccine dose strategies and variant scenarios. We assume AZD1222 is implemented for the first 2 doses, mRNA-1273 for the first booster (dose 3), and a variant-adapted vaccine for subsequent booster doses (doses 4 and 5). Table I. Total doses delivered, infections, hospitalisations, and deaths for the Category 2 setting, for a range of vaccine dose strategies and variant scenarios. We assume no additional variant emergence beyond Omicron (i.e., constant transmission and no additional immune escape, or no “drift”). Table J. Total doses delivered, infections, hospitalisations, and deaths for the Category 2 setting, for a range of vaccine dose strategies and variant scenarios, assuming the WHO coverage target assumption. We assume AZD1222 is implemented for the first 2 doses, mRNA-1273 for the first booster (dose 3), and a variant-adapted vaccine for subsequent booster doses (doses 4 and 5). Table K. Total additional infections, hospitalisations, and deaths averted, and total additional vaccine doses delivered for the Category 1 and 3 settings. We assume no additional variant emergence beyond Omicron (i.e., constant transmission and no additional immune escape, or no “drift”). Table L. Total additional infections, hospitalisations, and deaths averted, and total additional vaccine doses delivered for the Category 2 setting. We assume no additional variant emergence beyond Omicron (i.e., constant transmission and no additional immune escape, or no “drift”). Table M. Total doses delivered, infections, hospitalisations, and deaths for the Category 2 setting, for a range of vaccine dose strategies, for the scenario where individuals 40+ years are initially targeted. We assume AZD1222 is administered for all doses. Table N. Total doses delivered, infections, hospitalisations, and deaths for different ancestral, variant-adapted, and yearly updated vaccine scenarios. Table O. Impact of vaccination in future scenarios where an additional variant of concern emerges from 1 October 2023.
(DOCX)

## Abbreviations

COVID-19: Coronavirus Disease 2019
HIC: high-income country
ICU: intensive care unit
IL: immunity level
LMIC: lower-middle-income country
SARS-CoV-2: Severe Acute Respiratory Syndrome Coronavirus 2
VFR: variant fold reduction
WHO: World Health Organization
