# Supplementary material for: Long-term vaccination strategies to mitigate the impact of SARS-CoV-2 transmission: A modelling study
Source: PLoS Med. 2023 Nov 28;20(11):e1004195. doi: 10.1371/journal.pmed.1004195 (PMC10715640; doi:10.1371/journal.pmed.1004195)
Supplement: S1 Text — Fig A. Schematic diagram illustrating the COVID-19 vaccine allocation algorithm. Fig B. Modelled trajectories of the reproduction number Rt over time. Fig C. Exemplar demographic patterns for each of the 2 income settings. Fig D. Impact of vaccination in a high-income country setting with substantial prior transmission and high vaccine access, assuming no additional variant emergence beyond Omicron (i.e., constant transmission and no additional immune escape, or no “drift”). Fig E. Impact of vaccination in a lower-middle-income country setting with substantial prior transmission and moderate vaccine access, assuming no additional variant emergence beyond Omicron (i.e., constant transmission and no additional immune escape, or no “drift”). Fig F. Impact of vaccination in a high-income country setting with minimal prior transmission and high vaccine access (Category 3). We assume mRNA-1273 is implemented for the first 2 doses and the first booster (dose 3) and a variant-adapted vaccine for subsequent booster doses. Fig G. Impact of vaccination in a low-middle-income country setting with substantial prior transmission and low vaccine access, where individuals 40+ years are initially targeted. Fig H. Impact of vaccination in a lower-middle-income country setting with substantial prior transmission and moderate vaccine access (Category 2), assuming WHO coverage targets. Fig I. Comparison of vaccine impact for different ancestral and variant-adapted vaccine scenarios for the lower-middle-income country setting with substantial prior transmission (Category 2). Fig J. Comparison of vaccine impact for different ancestral and variant-adapted vaccine scenarios for the high-income country setting with minimal prior transmission (Category 3). Fig K. Impact of vaccination in future scenarios where an additional variant of concern emerges from 1 October 2022, in a high-income setting with minimal prior transmission and high vaccine access (Category 3). We assume a variant-adapted [file pmed.1004195.s001.docx]

**Supplementary material**

**Long term vaccination strategies to mitigate the impact of SARS-CoV-2 transmission: a modelling study**

Alexandra B Hogan^1,2*^, Sean L Wu^3^, Jaspreet Toor^2^, Daniela Olivera Mesa^2^, Patrick Doohan^2^, Oliver J Watson^2,4^, Peter Winskill^2^, Giovanni Charles^2^, Gregory Barnsley^2^, Eleanor M Riley^5^, David S Khoury^6^, Neil M Ferguson^2^, Azra C Ghani^2*^

1. School of Population Health, Faculty of Medicine and Health, University of New South Wales, Sydney, Australia
2. MRC Centre for Global Infectious Disease Analysis, Jameel Institute, School of Public Health, Imperial College London, London, UK
3. Institute for Health Metrics and Evaluation, University of Washington, Seattle, USA
4. London School of Hygiene and Tropical Medicine, London, UK
5. Institute of Immunology and Infection Research, School of Biological Sciences, University of Edinburgh, UK
6. Kirby Institute, University of New South Wales, Sydney, Australia

# S1. Supplementary methods

## S1.1 Immunological model

To capture the dynamics of vaccine-induced and infection-induced protection, we apply an existing within-host theoretical model of the relationship between immunity level (IL) over time, and protection against mild disease (which we assume to be the same as protection against any infection) and against severe disease requiring hospitalisation [1–3]. The approach is fully described in Hogan *et al.* (2023) [3] and is described briefly as follows. First, we model each individual’s IL over time, which is based on a biphasic exponential decay function,

$$n(t)=n_{ij}\frac{exp\left( \pi_{1}t+\pi_{2}t_{s} \right)+exp\left( \pi_{2}t+\pi_{1}t_{s} \right)}{exp\left( \pi_{1}t_{s} \right)+exp\left( \pi_{2}t_{s} \right)},$$

where $n_{ij}$ is the initial IL of vaccine *i* drawn from a log_10_-normal distribution at dose *j*; $\pi_{1}=-ln\left( 2 \right)/h_{s}$ is the rate for the initial period of fast antibody decay; $\pi_{2}=-ln\left( 2 \right)/h_{l}$ is the rate for the period of slow decay; $t$ is the time since the last dose or infection; and $t_{s}$ is the period of switching between the fast and slow decays.

We then assume a logistic relationship between IL and effectiveness,

$$\epsilon_{m}\left( n_{t} \right)=\frac{1}{1+e^{-k[{log}_{10}\left( n_{t} \right)-{log}_{10}\left( n_{{50}_{m}} \right)]}},$$

where $k$ is the fitted shape parameter and $n_{{50}_{m}}$ is the IL relative to convalescents required to provide 50% protection against each endpoint (infection ($m=1)$ and hospitalisation ($m=2)$).

Khoury *et al* parameterised this model using efficacy data from Phase III trials, where the reported neutralizing antibody titre (NAT) data following dose 2 was expressed as relative to the mean titre for a convalescent individual [1]. By definition, on this scale, the NAT induced by infection in convalescent individuals is 1. To capture infection-induced immunity, we use the same approach, assuming that each infection generates a boost to the level of IL observed in convalescent individuals (which is scaled to 1).

The fitted immunological parameters (from Hogan *et al* 2023 [3]) are summarised in Table A.

**Table A:** **Prior and posterior parameter estimates for the immunological model (including fitted parameters from Hogan et al. 2023** [3]**).** IL: immunity level; VFR: variant fold reduction

| Parameter | Symbol | Posterior estimate for  median (95% credible interval) |
| --- | --- | --- |
| **Immunity levels for each vaccine** | | |
| Oxford/AstraZeneca AZD1222 vaccine | | |
| IL against Delta for dose 1 relative to convalescent | $n_{AZ,1}$ | 0.10 (0.14, 0.08) |
| IL against Delta for dose 2 relative to convalescent | $n_{AZ,2}$ | 0.36 (0.29, 0.45) |
| IL against Delta for dose 3 relative to convalescent | $n_{AZ,3}$ | 0.60 (0.45, 0.82) |
| Pfizer-BioNTech BNT162b2 vaccine | | |
| IL against Delta for dose 1 relative to convalescent | $n_{PF,1}$ | 0.30 (0.24, 0.38) |
| IL against Delta for dose 2 relative to convalescent | $n_{PF,2}$ | 0.69 (0.55, 0.87) |
| IL against Delta for dose 3 relative to convalescent | $n_{PF,3}$ | 0.92 (0.71, 1.19) |
| Moderna mRNA-1273 vaccine | | |
| IL against Delta for dose 1 relative to convalescent | $n_{MD,1}$ | 0.20 (0.16, 0.27) |
| IL against Delta for dose 2 relative to convalescent | $n_{MD,2}$ | 0.93 (0.72, 1.20) |
| IL against Delta for dose 3 relative to convalescent | $n_{MD,3}$ | 1.13 (0.86, 1.49) |
| Immune escape parameters | | |
| Fold reduction for Omicron relative to Delta (all vaccines) | VFR | 5.1 (4.0, 6.8) |
| Fold reduction scaling factor for Omicron (BA.1/BA.2) relative to Delta assuming a variant-adapted vaccine | $\psi$ | **0.62** |
| Immunity level decay parameters | | |
| Half-life of IL decay: short (days) | $h_{s}$ | 35 (31, 38) |
| Half-life of IL decay: long (days) | $h_{l}$ | 581 (354, 872) |
| Time period for switching (days) | $t_{s}$ | 75 (60, 88) |
| Relationship between IL and protection | | |
| IL relative to convalescent required to provide 50% protection from mild disease | ${n_{50}}_{1}$ | 0.091 (0.066, 0.125) |
| IL relative to convalescent required to provide 50% protection from hospitalisation | ${n_{50}}_{2}$ | 0.021 (0.012, 0.035) |
| IL relative to convalescent required to provide 50% protection from death | ${n_{50}}_{3}$ | 0.021 (0.012, 0.036) |
| NAT relative to convalescent for protection against re-infection | $n_{I}$ | 1 (by definition) |
| NAT scaling for strain-specific protection against re-infection following variant emergence | ξ | 0.5 |
| Shape parameter | $k$ | 3.1 (2.7, 3.7) |
| Standard deviation of individual responses | - | 0.44 (from Khoury *et al* [1]) |

## S1.2 Population model

We developed an individual-based population model of transmission (“*safir*”) to explore the long-term dynamics of SARS-CoV-2 transmission. The model framework builds upon previous compartmental models to capture transmission and the allocation of vaccines [3,4].

Infection is seeded into the population at the start of the model simulation (1 February 2020). The simulation uses a discrete time step update of 0.25 days and is run for a population size of 1 million individuals.

The main model captures the infection status of individuals as being in one of the following states (Fig 1 in the main text):

- S = uninfected and therefore **susceptible** to infection
- E = **exposed** to infection but not yet infectious
- I_mild_ = **infected** and infectious with mild symptomatic infection that does not require hospitalisation
- I_asymp_ = **infected** and infectious with asymptomatic infection
- I_case_ = **infected** and infectious with disease that will require hospitalisation
- I_hosp_ = cases that have been **hospitalised** in a general ward bed
- I_ICU_ = cases that have been admitted to **an intensive care unit (ICU)**
- I_rec_ = cases that have been stepped down from ICU into a general ward bed for **recovery**
- D = cases that have **died**

The transmission model is identical to the compartmental structure described in Hogan et al 2021 [5], with the inclusion of a separate state to distinguish asymptomatic infection from mild symptomatic infection (Fig 1 in the main text). Furthermore, we additionally track the IL for each individual (see below for further details) which determines their risk of becoming infected and subsequent risk of disease progression. To decrease the probability of stochastic fadeout, we additionally include a constant external (unmodelled) force of infection, which is added to each susceptible individual’s force of infection, before calculating the protection against infection from that individual’s IL. This external force of infection represents external infection pressure from outside the modelled population. Finally, because we track IL as a measure of protection, the recovered state is no longer required, and individuals return to the susceptible state with an increased level of immunity.

Following identical notation to Hogan et al 2021 [5], the underlying transmission of SARS-CoV-2 is represented by the set of events described in Table B. All state durations are modelled using an Erlang distribution with a shape parameter of 2.

**Table B: Transmission model state transitions. ICU: intensive care unit**

| **Event** | **State transition** | **Transition rate** |
| --- | --- | --- |
| New infection | S → E | $\lambda\left( a,n,t \right)+\lambda_{e}$ |
| Latent to asymptomatic | E → I_asymp_ | $\phi_{0}\left( a \right)(1-\phi_{1}\left( a,n \right))\alpha$ |
| Latent to mild symptomatic | E → I_mild_ | $(1-\phi_{0}\left( a \right))(1-\phi_{1}\left( a,n \right))\alpha$ |
| Latent to severe | E → I_case_ | $\phi_{1}\left( a,n \right)\alpha$ |
| Severe case to ICU | I_case_ → I_ICU_ | $\phi_{2}\left( a \right)\gamma_{2}$ |
| Severe case to hospital | I_case_ → I_hosp_ | $\left( 1-\phi_{2}\left( a \right) \right)\gamma_{2}$ |
| ICU to death | I_ICU_ → D | ${\mu_{2}(a)\gamma}_{4,0}$ |
| Hospitalised to death | I_hosp_ → D | ${\mu_{1}(a)\gamma}_{3}$ |
| ICU to ICU recovered | I_ICU_ → I_rec_ | $(1-\mu_{2}\left( a \right))\gamma_{4,1}$ |
| ICU recovered to susceptible | I_rec_ → S | $\gamma_{5}$ |
| Hospitalised to susceptible | I_hosp_ → S | $(1-\mu_{1}\left( a \right))\gamma_{3}$ |
| Symptomatic case to susceptible | I_case_ → S | $\gamma_{1}$ |
| Asymptomatic case to susceptible | I_asymp_ → S | $\gamma_{1}$ |

We additionally incorporate a switch when hospital capacity is exceeded. Those who cannot be hospitalised experience a non-age-dependent higher death rate which differs depending on whether they would have required ICU. The parameters are summarised in Table C.

The force of infection $\lambda\left( a,n,t \right)$ depends on a setting-specific age-structured contact matrix, $c\left( a,a^{'} \right)$ [4]. We assume that only infections in the community contribute to onward infection (i.e. there is no nosocomial transmission) and therefore:

$$\lambda\left( a,n,t \right)=\beta_{t}(1-\epsilon_{1}\left( n_{t} \right))\sum_{a^{'}} c\left( a,a^{'} \right)[I_{asympt}\left( a^{'},t \right)+I_{mild}(a^{'},t)+ I_{case}(a^{'},t)].$$

ILs over time, denoted as $n(t)$, are computed on a log_10_ scale and altered by vaccination and infection. Total IL is the sum of vaccine-induced and infection-induced IL on the log_10_ scale:

$$n\left( t \right)=n_{V}\left( t \right)+n_{I}\left( t \right).$$

Two boosting events can occur. Firstly, vaccination results in an increase in vaccine-induced IL:

$$n_{V}\left( t \right)\to n_{V}\left( t \right)+\mathcal{N}\left( n_{v,d},\sigma^{2} \right),$$

where $n_{v,d}$ is the mean boost associated with vaccine $v$ and dose $d$and $\sigma$ is the standard deviation between individuals (see Table A). Subsequent vaccine doses are correlated within individuals, meaning that subsequent boosts from doses are equal to the random initial draw multiplied by the ratio of the previous mean dose-specific IL to this dose-specific value. This allows us to implicitly capture individual level heterogeneity in immune response to vaccination.

Secondly, on recovering from infection (i.e. at the same time that the individual returns to the susceptible state S), infection-induced ILs are boosted:

$$n_{I}\left( t \right)\to n_{I}\left( t \right)+\mathcal{N}\left( n_{I},\sigma^{2} \right),$$

where $n_{I}$ is the fixed boost associated with a past infection and $\sigma$ is the standard deviation between individuals.

Each person’s IL decays linearly on a natural log scale, as described above. ILs from infection are assumed to decay at the same rate as ILs following vaccine doses. Each exposure or vaccine dose results in an additive increase in IL. We imposed an upper limit of $\text{exp}(5.0)$ on a linear scale for the total vaccine- or infection-induced IL an individual can achieve to represent an arbitrary upper biological limit, given studies that indicate a maximum level of immunogenicity following vaccination [6].

An individual’s level of immunity is used to calculate protection as described above (capturing infection-induced immunity and vaccine-induced immunity in combination). We model protection against infection by reducing the probability that susceptible individuals transition to exposed ($\epsilon_{1}\left( n \right)$) and protection against severe disease by reducing the probability of developing severe disease requiring hospitalisation:

$$\phi_{1}\left( a,n \right)={(1-\epsilon}_{2}\left( n \right))\phi_{1}\left( a \right).$$

**Table C: Transmission parameter description and values (from Hogan *et al* 2021** [5]**).** ICU: intensive care unit; IFR: infection fatality ratio; NAT: neutralizing antibody titre; R: reproduction number

| **Parameter** | **Symbol** | **Value** | **Description** |
| --- | --- | --- | --- |
| **Epidemiological Parameters** | | | |
| Transmission parameter | $\beta_{t}$ | - | Calculated from R |
| External infection source | $\lambda_{e}$ | 1e-7 per day | Low-level external source of infection to prevent stochastic fadeout |
| Mean latent period | $\frac{1}{\alpha}$ | 4.6 days | Estimated at 5.1 days. The last 0.5 days are incorporated in the infectious periods to capture pre-symptomatic infectivity |
| Mean duration of mild or asymptomatic infection | $\frac{1}{\gamma_{1}}$ | 2.1 days | Incorporates 0.5 days of infectiousness prior to symptoms. In combination with mean duration of severe illness this gives a mean serial interval of 6.75 days. |
| Mean duration of severe infection prior to hospitalisation | $\frac{1}{\gamma_{2}}$ | 4.5 days | Mean onset-to-admission of 4 days. Includes 0.5 days of infectiousness prior to symptom onset. |
| Mean duration of hospitalisation for non-critical cases if survive | $\frac{1}{\gamma_{3}}$ | 9 days | - |
| Mean duration in ICU if survive | $\frac{1}{\gamma_{4,1}}$ | 14.8 days | Mean duration in ICU of 13.3 days. Ratio of duration in critical care if die: duration in critical care if survive of 0.75 and 60.1% probability of survival in ICU. |
| Mean duration in ICU if die | $\frac{1}{\gamma_{4,0}}$ | 11.1 days | Mean duration in ICU of 13.3 days. Ratio of duration in critical care if die: duration in critical care if survive of 0.75 and 60.1% probability of survival in ICU. |
| Mean duration in recovery after ICU | $\frac{1}{\gamma_{5}}$ | 3.0 days | Assumption |
| IFR in the absence of NAT from non-ICU | $\mu_{1}(a)$ | - | Age-dependent (see Table S2 in Hogan *et al* 2021[5]) |
| IFR in the absence of NAT from ICU | $\mu_{2}\left( a \right)$ | - | Age-dependent (see Table S2 in Hogan *et al* 2021 [5]) |
| IFR for non-ICU if no hospital bed available |  | 0.6 | Assumption as in Walker *et al* [4] |
| IFR for ICU if no ICU available | - | 0.95 | Assumption as in Walker *et al* [4] |
| Proportion of mild infections that are asymptomatic | $\phi_{0}(a)$ | 0.2 | - |
| Probability of developing severe disease requiring hospitalisation in the absence of NAT | $\phi_{1}(a)$ | - | Age-dependent (see Table S2 in Hogan *et al* 2021 [5]) |
| Proportion of hospitalisations requiring ICU | $\phi_{2}(a)$ | - | Age-dependent (see Table S2 in Hogan *et al* 2021 [5]) |
| Age-structured contact matrix | $c\left( a,a^{'} \right)$ | See Table E | Walker *et al* [4] |

## S1.3 Transmission

Modelled transmission is allowed to vary over time by setting a time-varying reproduction number, $R_{t}$, such that

$$\beta_{t}=\frac{R_{t}}{\vartheta},$$

where $\vartheta$ is the dominant eigenvalue of the next generation matrix:

$$\boldsymbol{A}=c\left( a,a^{'} \right)\left[ \frac{\phi_{0}\left( a \right)(1-\phi_{1}\left( a \right))}{\gamma_{1}}+\frac{{(1-\phi}_{0}\left( a \right))(1-\phi_{1}\left( a \right))}{\gamma_{1}}+\frac{\phi_{1}\left( a \right)}{\gamma_{2}} \right].$$

## S1.4 Capturing the effect of Omicron, subsequent variants, and variant-adapted vaccines

To model the impact of the Omicron variant, we introduce Omicron on 27/11/2021 and linearly scale up its presence to 100% by 31/12/2021 whilst concomitantly decreasing the presence of the Delta variant to 0% by this date. Rather than explicitly simulate the two variants, we capture this impact by modifying the immunity in the population as well as the severity of the virus. We assume no change in transmissibility as our estimated immune escape results in patterns that are consistent with the observed trends in countries without any increase in intrinsic transmissibility.

To capture the reduced effectiveness of vaccines against the Omicron variant, we linearly reduce the vaccine-induced IL (denoted by $n_{V}^{i}\left( t \right),$ where $i$ represents the vaccine dose) over this period such that by 31/12/2021 the IL have been reduced by the variant fold reduction (VFR) – and hence the vaccine effectiveness has been reduced to the levels estimated in Table A. Mathematically:

$$\begin{matrix} n_{V}^{i}\left( t \right)=n_{V}\left( t \right) & t \leq t_{1}, \\ n_{V}^{i}\left( t \right)= \frac{\left( t-t_{1} \right)}{\left( t_{2}-t_{1} \right)}n_{V}\left( t \right)+\frac{{(t}_{2}-t)}{(t_{2}-t_{1})}\frac{n_{V}(t)}{VFR} & t_{1}<t<t_{2,} \\ n_{V}^{i}\left( t \right)= n_{V}\left( t \right)/VFR & t \geq t_{2}, \end{matrix}$$

where $t_{1}$ is 27/11/2021 and $t_{2}$ is 31/12/2021.

This same VFR is then applied to all additional booster doses using the ancestral vaccines such that:

$$n_{V}^{i+1}\left( t \right)=n_{V}^{i}\left( t \right)+\mathcal{N}\left( n_{v,d}/VFR,\sigma^{2} \right) t \geq t_{2}.$$

For booster doses administered with a variant-adapted vaccine (rather than an ancestral vaccine), we reduce the impact of the VFR, thereby increasing the effectiveness level such that

$$n_{V}^{i+1}\left( t \right)=n_{V}^{i}\left( t \right)+\mathcal{N}\left( n_{v,d}\frac{\psi}{VFR},\sigma^{2} \right) t \geq t_{2},$$

where $\frac{VFR}{\psi}$ represents the scaled VFR to generate the additional effectiveness of the variant-adapted vaccine.

To capture the impact of immune escape on infection-induced immunity, we similarly apply the VFR to the infection-induced IL (denoted by $n_{I}^{j}\left( t \right),$ where $j$ represents the infection number) prior to 27/11/2021 (which we assume to be driven by Delta or previous variants) and to the proportion of Delta variants assumed to be circulating between 27/11/201 and 31/12/2021 for infections that occur during this period. Thus

$$\begin{matrix} n_{I}^{j}\left( t \right) =n_{I}\left( t \right) & t \leq t_{1}, \\ n_{I}^{j}\left( t \right) = \frac{\left( t-t_{1} \right)}{\left( t_{2}-t_{1} \right)}n_{I}\left( t \right)+\frac{{(t}_{2}-t)}{(t_{2}-t_{1})}\frac{n_{I}(t)}{VFR} & t_{1}<t<t_{2,} \\ n_{I}^{j}\left( t \right) = n_{I}\left( t \right)/VFR & t \geq t_{2}. \end{matrix}$$

However, in contrast to the vaccines, the VFR is not applied to the IL boost from the proportion of infections assumed to be due to Omicron between 27/11/2021 and 31/12/2021 nor to any infections occurring from 01/01/2022 onwards which are assumed to be due to Omicron, with a scaling factor introduced in order to capture strain-specific protection against infection. Thus

$$n_{I}^{j+1}\left( t \right) =\zeta n_{I}^{j}\left( t \right)+\mathcal{N}\left( n_{I},\sigma^{2} \right) t \geq t_{2}$$

Finally, we modelled a linear decrease in severity of all infections occurring over the same time period, reducing the probability of requiring hospitalisation by 70% and the probability of severe disease (requiring ICU and conditional on hospitalisation) by an additional 70% [7]. This reduced severity is then applied to all infections from 01/01/2022 onwards.

For the analyses in which a new variant is introduced, the same process is applied (using the parameters described in the main text to modify immune escape and/or severity). The VFR change is either applied between 01/10/2023 and 31/10/2023 (for the analysis of a single dominant variant replacing Omicron) or is applied as an incremental step-change increase every 4 months starting from 01/04/2022. We additionally incorporate a linear change in transmissibility for these scenarios over the same time window as the VFR increase, which is applied directly to the R_t_ transmission profile.

## S1.5 Vaccine allocation

For each dose, we specify a matrix of coverage targets by age-group (columns) where rows are ordered prioritisation steps. Elements of the matrix thus identify for each step what target percentage dose coverage of that age-group needs to be fulfilled. We identify what dose is being distributed by distribution “phase” (e.g. on phase 2, the second dose is being distributed). Within that distribution phase, the coverage target matrix specific to that dose/phase is used to distribute doses (subject to availability) according to each row of the matrix. When all coverage targets of a particular prioritisation step (a row) are fulfilled, the distribution algorithm moves to the next step (i.e. the next row). When coverage targets for all steps in a particular dose are achieved, we may move on to the next dose phase (i.e. distribute the next dose).

On each day for which there are at least some available doses, the algorithm checks which priority step in a phase it is on. If a phase is completed, it advances to the next phase. If not, based on the targets for that step, the set of eligible persons to receive that dose is identified. This considers which age-groups have not yet met coverage targets, and which persons have had an adequate waiting period since their last dose (if phase > 1). Available doses are first distributed to prioritised persons who may receive the next dose based on if the minimum waiting period between doses has been met (e.g. if the current phase is distributing dose 1, we first distribute dose 2 to prioritised age groups). Remaining doses are then distributed to persons eligible for this dose, beginning with the current priority step. The algorithm distributes to eligible persons by age group, walking down the list of priority steps until either no doses remain, or all steps are exhausted. The algorithm stops if all targets for all doses are filled. The flowchart in Fig A describes how the algorithm functions on a single day.


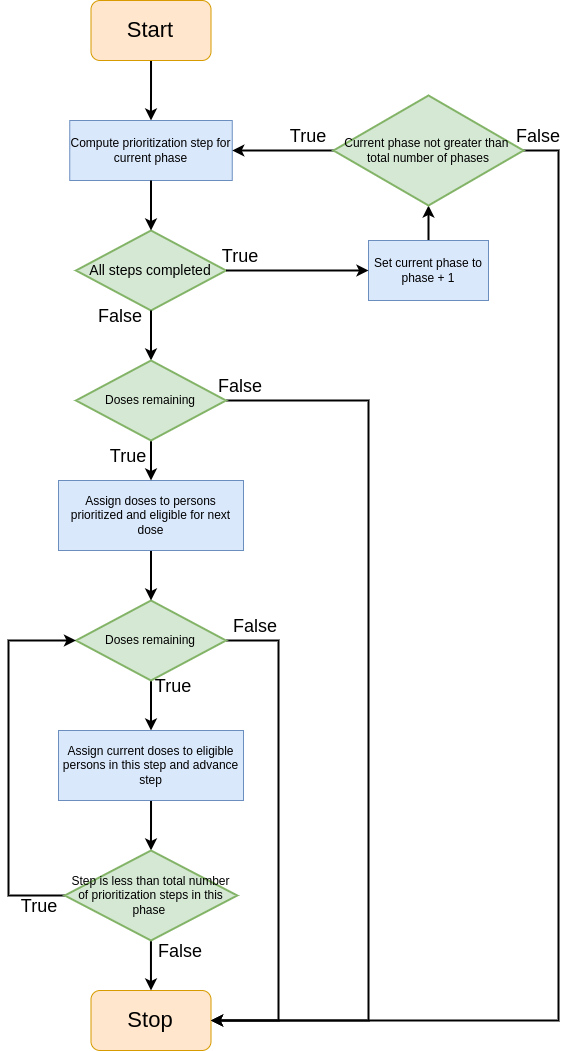


**Fig A: Schematic diagram illustrating the COVID-19 vaccine allocation algorithm.**

## S1.6 Transmission scenarios

Because the types and impacts of non-pharmaceutical interventions vary depending on setting, rather than modelling any specific non-pharmaceutical interventions (such as social distancing measures, mask-wearing, or school closures), we assume that the impact of these measures is reflected in the reproduction number. For the country settings in Categories 1 and 2 (countries with substantial prior transmission before the rollout of vaccination), we construct a representative trajectory of transmission by varying R_t_ for the Delta variant to match patterns observed in these settings (guided by country-level fits from our global model fitting – see <https://mrc-ide.github.io/global-lmic-reports> and [8]), such that an initial epidemic wave occurs between March and May 2020, a second wave occurs during December 2020 and January 2021. For all settings we model a third wave (the Delta wave) occurring approximately from September onwards (similar to that experienced in most European countries) with the resulting R_t_ for Delta reaching 4 while in the lower-middle-income (LMIC) countries we allow a more rapid increase from mid-2021 to capture the earlier Delta waves. For the high-income country (HIC) settings in Category 3 (countries with minimal prior transmission before the rollout of vaccination), we construct a representative trajectory such R_t_ < 1 (no sustained transmission) until September 2021, at which point R_t_ increases gradually to 4 by end-2022. For all three categories, we assume that the virus continues to evolve or “drift” over time, with a new variant regularly replacing the dominant variant. This this is reflected in the level of transmission by increasing R_t_ every 4 months. Fig B shows the resulting transmission profile for each category.


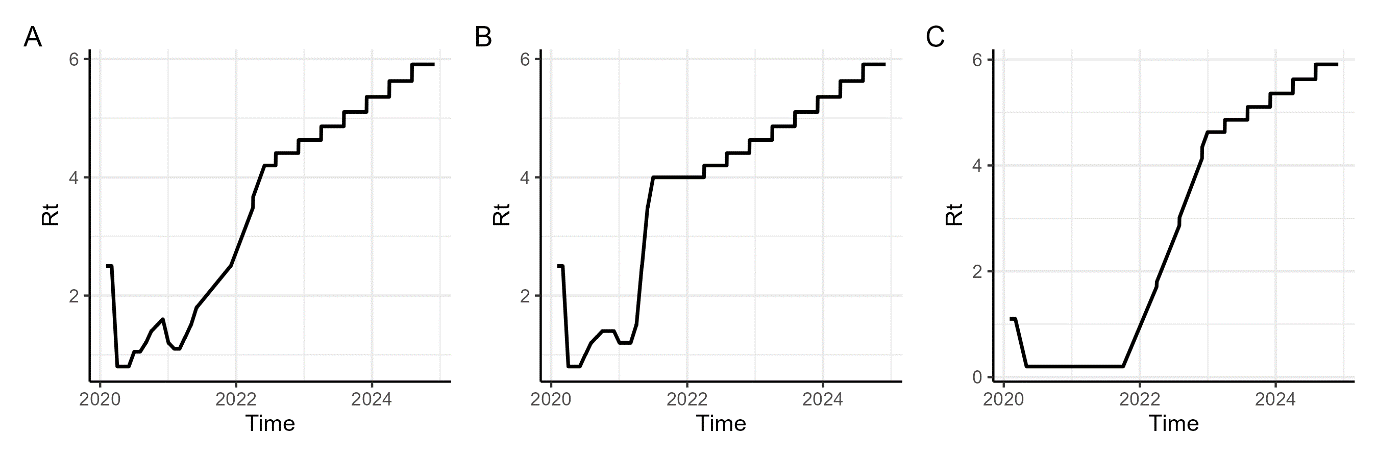


**Fig B: Modelled trajectories of the reproduction number R_t_ over time**. (A) the high-income country (HIC) setting with substantial prior transmission (Category 1); (B) the lower-middle-income country (LMIC) setting with substantial prior transmission (Category 2); and (C) the HIC setting with minimal prior transmission (Category 3).

## S1.7 Vaccine coverage

Vaccine coverage assumptions for each of the income settings are summarised in Table D and Fig C.

**Table D: Default scenarios and assumptions for vaccine uptake within targeted age groups for each of the two income settings.** Total future maximum population-level coverage for each income setting is based on the World Health Organization (WHO) current reported coverage by income setting, which on 20 June 2023 reported 75% and 48% for the primary series and booster doses respectively in high-income country (HIC) settings, and 61% and 19% respectively in lower-middle-income country (LMIC) settings [9]. The “LMIC – higher coverage” scenario is based on the stated WHO target of 70% total population coverage by mid-2022 [10], which we apply to 10+ years age groups.

| Age group (years) | Vaccine uptake | | | | | |
| --- | --- | --- | --- | --- | --- | --- |
|  | HIC | | LMIC – default | | LMIC – higher coverage | |
|  | Primary series | Booster doses | Primary series | Booster doses | Primary series | Booster doses |
| 0-4 | 0% | 0% | 0% | 0% | 0% | 0% |
| 5-9 | 0% | 0% | 0% | 0% | 0% | 0% |
| 10-14 | 60% | 20% | 75% | 49% | 87% | 57% |
| 15-19 | 60% | 20% | 75% | 49% | 87% | 57% |
| 20-24 | 80% | 30% | 75% | 49% | 87% | 57% |
| 25-29 | 80% | 30% | 75% | 49% | 87% | 57% |
| 30-34 | 80% | 30% | 75% | 49% | 87% | 57% |
| 35-39 | 80% | 60% | 75% | 49% | 87% | 57% |
| 40-44 | 90% | 60% | 75% | 49% | 87% | 57% |
| 45-49 | 90% | 60% | 75% | 49% | 87% | 57% |
| 50-54 | 90% | 60% | 75% | 49% | 87% | 57% |
| 55-59 | 90% | 60% | 75% | 49% | 87% | 57% |
| 60-64 | 90% | 80% | 75% | 49% | 87% | 57% |
| 65-69 | 90% | 80% | 75% | 49% | 87% | 57% |
| 70-74 | 90% | 80% | 75% | 49% | 87% | 57% |
| 75-79 | 90% | 80% | 75% | 49% | 87% | 57% |
| 80+ | 90% | 80% | 75% | 49% | 87% | 57% |
| Population-level (all ages) | 80% | 53% | 56% | 39% | 70% | 45% |


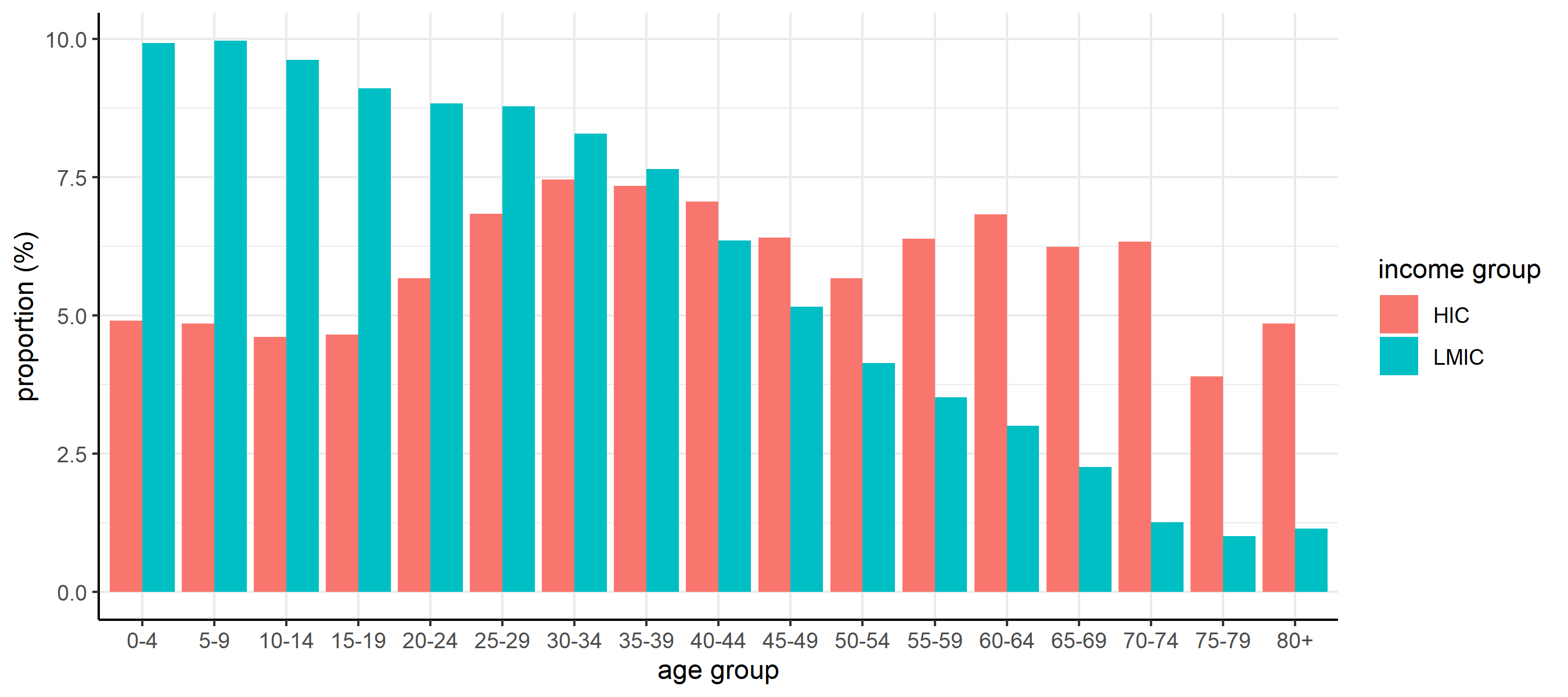


**Fig C: Exemplar demographic patterns for each of the two income settings**. HIC: high-income country; LMIC: lower-middle-income country

## S1.8 Summary of scenarios explored

Assumptions for the default scenarios explored, for the three modelled categories of countries and epidemiological state, are summarised in Table E and Fig 2 of the main text.

**Table E: Default scenarios and assumptions for the three broad modelled categories of country and epidemiological state.** For each of the two income settings (high-income country (HIC) and lower-middle-income country (LMIC)), we use the current age distribution and estimated health system capacity for the country with the median GDP within that income group, and we further use a representative contact pattern for a country within that income group based on the availability of contact survey data, following the methodology of Walker *et al* [4] ICU: intensive care unit.

|  |  | **Category 1; Category 3** | **Category 2** |
| --- | --- | --- | --- |
| Setting | Income setting | High-income (HIC) | Lower-middle-income (LMIC) |
|  | Health system constraints | Unconstrained (no limit on number of hospital and ICU beds) | Constraints present |
|  | General beds available | 1 million | 800 |
|  | ICU beds available | 1 million | 16 |
|  | Contact patterns | HIC exemplar  <https://mrc-ide.github.io/squire/>  squire::get_mixing_matrix(country = “United Kingdom”) | LMIC exemplar  <https://mrc-ide.github.io/squire/>  squire::get_mixing_matrix(country = “Zimbabwe”) |
|  | Demography | HIC median  <https://mrc-ide.github.io/squire/>  squire::get_population(country = “Malta”) | LMIC median  <https://mrc-ide.github.io/squire/>  squire::get_population(country = “Nicaragua”) |
|  | Transmission | See Fig B | See Fig B |
| Vaccine characteristics | Product | Default: mRNA-1273 (doses 1–3); variant-adapted (dose 4 onwards) | Default: AZD1222 (doses 1 and 2); mRNA-1273 (dose 3); variant-adapted (dose 4 onwards) |
|  | Effectiveness | See Table A | See Table A |
|  | Short-term durability | Fitted (see Table A) | Fitted (see Table A) |
|  | Long-term durability | Fitted (see Table A) | Fitted (see Table A) |
| Timing of roll-out and doses | Vaccine start date | 1 January 2021 | 1 April 2021 |
|  | Timing of dose 2 | 28 days post-dose 1 | 28 days post-dose 1 |
|  | Timing of first booster dose | 227 days post-dose 2 | 227 days post-dose 2 |
|  | Timing of subsequent booster doses | 180 days or 365 days post-dose 3 | 365 days post-dose 3 |
|  | Vaccination rate | 5% per week | 2% per week |
| Vaccine targeting | Maximum uptake: primary series | 80% (See Table D) | 56% (See Table D) |
|  | Maximum uptake: booster doses | 53% (See Table D) | 39% (See Table D) |
|  | Allocation | See Methods | See Methods |
| Time window simulated | Start date | 1 February 2020 | 1 February 2020 |
|  | End date | 31 December 2024 | 31 December 2024 |

# S2. Supplementary results


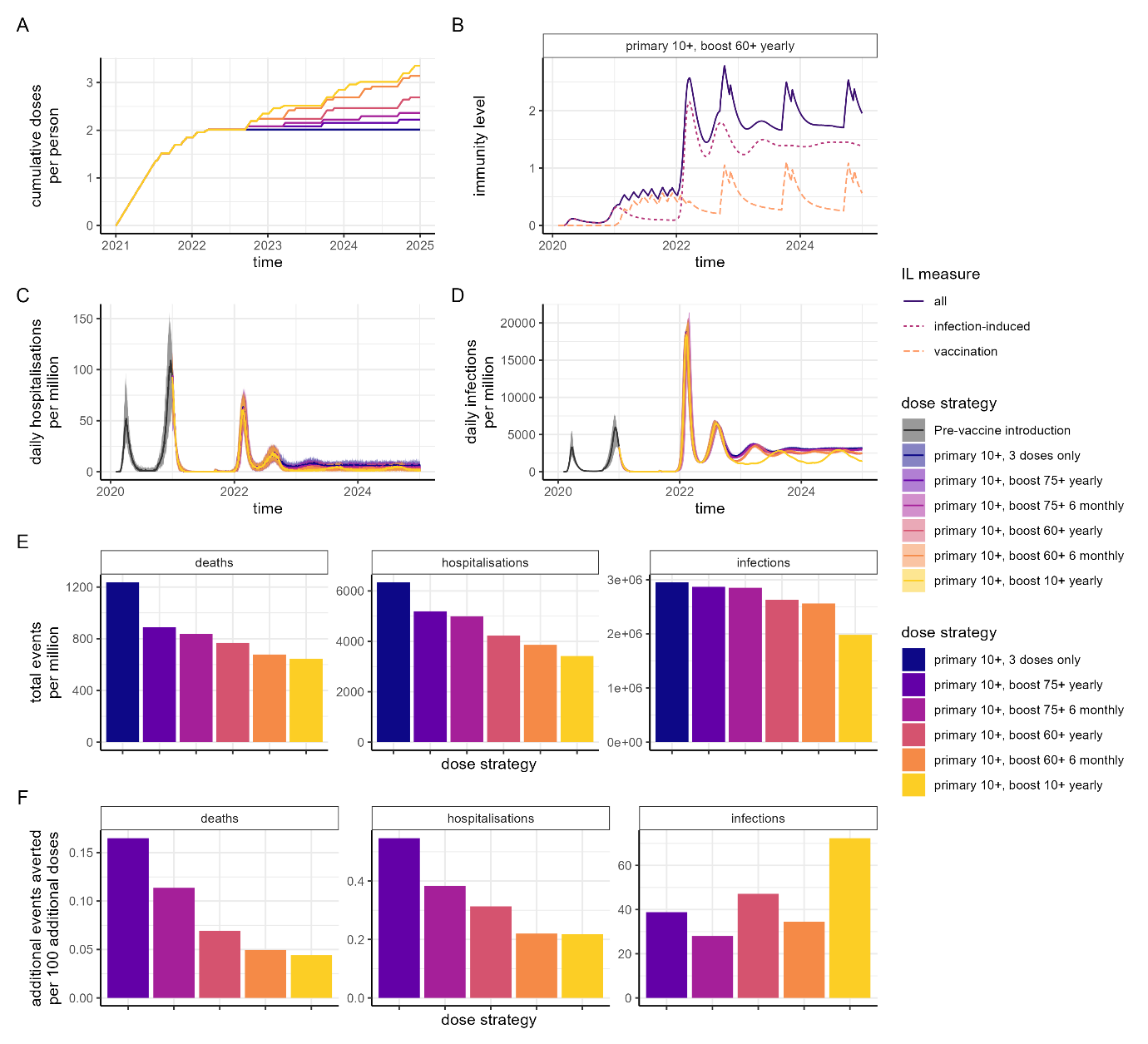


**Fig D: Impact of vaccination in a high-income country setting with substantial prior transmission and high vaccine access, assuming no additional variant emergence beyond Omicron (i.e constant transmission and no additional immune escape, or no “drift”).** We assume mRNA-1273 is implemented for the first 2 doses and the first booster (dose 3), and a variant-adapted vaccine for subsequent booster doses. (A) Cumulative doses delivered per person over time, for a range of dose delivery strategies. In all strategies, the primary series was delivered to individuals 10 years and older, with scenarios of no additional doses; annual or 6-monthly boosters to the 75+ years population; annual or 6-monthly boosters to the 60+ years population; or annual boosters to the 10+ years population. (B) Mean infection-induced (pink dotted), vaccine-induced (orange dashed), and total (purple solid) immunity level (IL) over time for the “primary 10+, boost 60+ yearly” dose strategy. (C) Daily hospitalisations and (D) daily infections per million population for the six dose strategies, where the trajectory prior to vaccine introduction is shown in dark grey. (E) Total events (deaths, hospitalisations, and infection) per million population between 1 July 2022 and 31 December 2024 for each dose strategy. (F) Additional events averted per 100 additional doses over the same time period relative to the “primary 10+, 3 doses only” dose strategy. Results for the default scenario with gradual viral drift (increasing transmission and immune escape) following the emergence of Omicron are shown in Fig 3 of the main text.


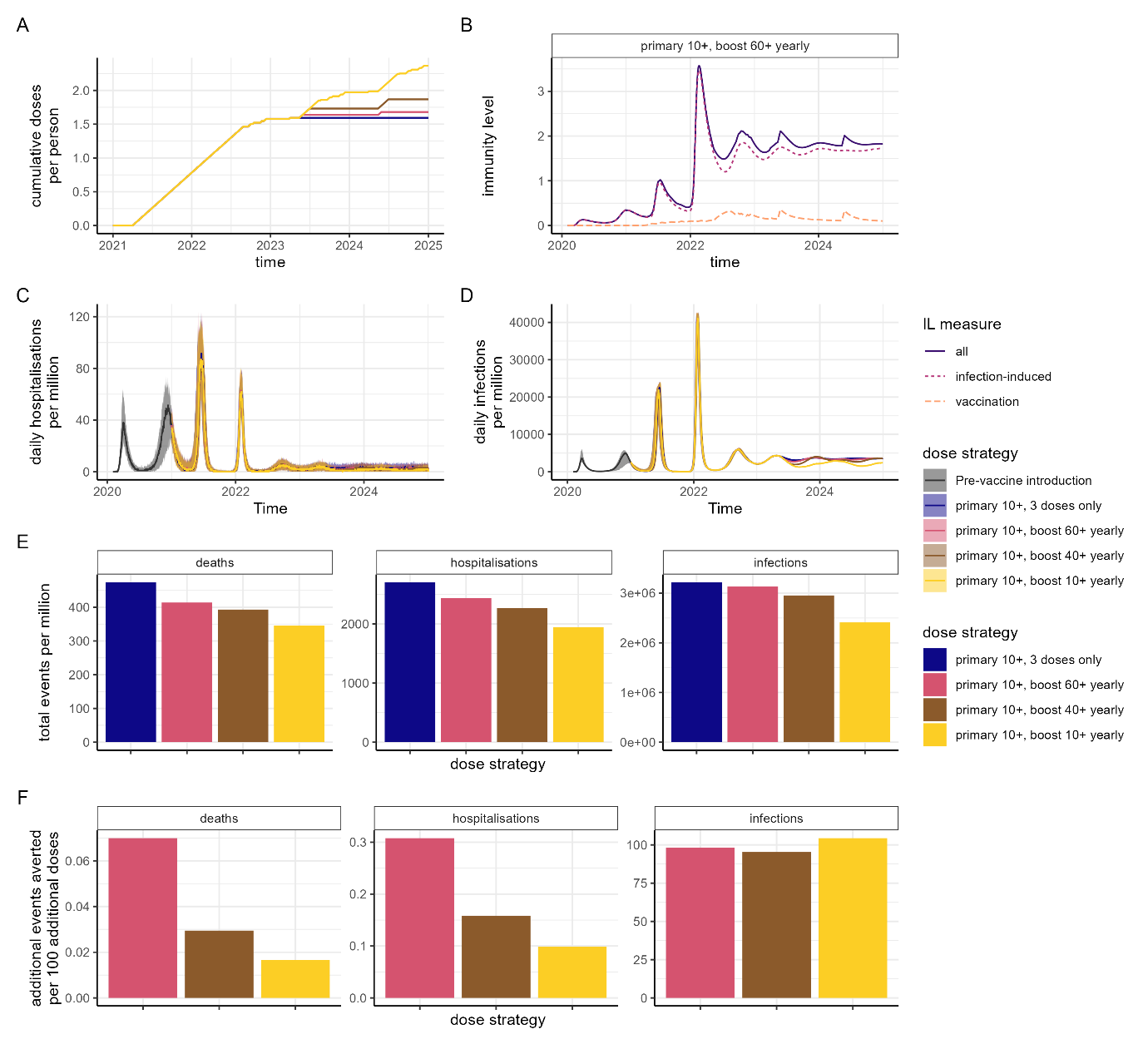


**Fig E: Impact of vaccination in a lower-middle-income country setting with substantial prior transmission and moderate vaccine access, assuming no additional variant emergence beyond Omicron (i.e constant transmission and no additional immune escape, or no “drift”).** We assume AZD1222 is implemented for the first 2 doses, and a variant-adapted vaccine for the booster doses. (A) Cumulative doses delivered per person over time, for a range of dose delivery strategies. In all strategies, the primary series was delivered to individuals 10 years and older, with scenarios of no additional doses; annual boosters to the 60+ years population; annual boosters to the 40+ years population; or annual boosters to the 10+ years population. (B) Mean infection-induced (pink dotted), vaccine-induced (orange dashed), and total (purple solid) immunity level (IL) over time for the “primary 10+, boost 60+ yearly” dose strategy. (C) Daily hospitalisations and (D) daily infections per million population for the dose strategies, where the trajectory prior to vaccine introduction is shown in dark grey. (E) Total events (deaths, hospitalisations, and infection) per million population between 1 July 2022 and 31 December 2024 for each dose strategy. (F) Additional events averted per 100 additional doses over the same time period relative to the “primary 10+, 3 doses only” dose strategy. Results for the scenario with gradual viral drift (increasing transmission and immune escape) following the emergence of Omicron are shown in Fig 4 of the main text.

**
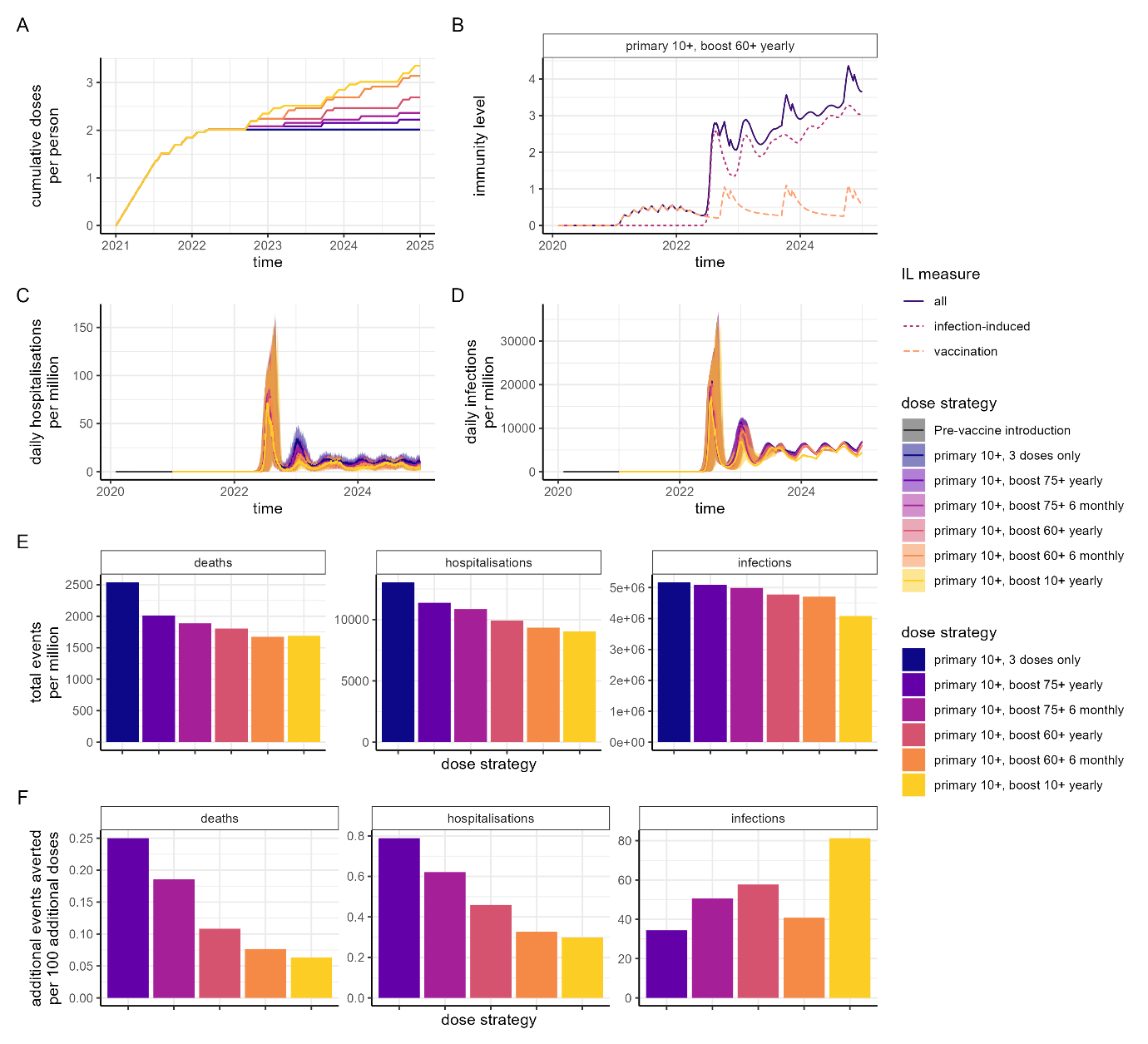
**

**Fig F: Impact of vaccination in a high-income country setting with minimal prior transmission and high vaccine access (Category 3). We assume mRNA-1273 is implemented for the first 2 doses and the first booster (dose 3), and a variant-adapted vaccine for subsequent booster doses.** (A) Cumulative doses delivered per person over time, for a range of dose delivery strategies. In all strategies, the primary series was delivered to individuals 10 years and older, with scenarios of no additional doses; annual or 6-monthly boosters to the 75+ years population; annual or 6-monthly boosters to the 60+ years population; or annual boosters to the 10+ years population. (B) Mean infection-induced (pink dotted), vaccine-induced (orange dashed), and total (purple solid) immunity level (IL) over time for the “primary 10+, boost 60+ yearly” dose strategy. (C) Daily hospitalisations and (D) daily infections per million population for the six dose strategies, where the trajectory prior to vaccine introduction is shown in dark grey. (E) Total events (deaths, hospitalisations, and infection) per million population between 1 July 2022 and 31 December 2024 for each dose strategy. (F) Additional events averted per 100 additional doses over the same period relative to the “primary 10+, 3 doses only” dose strategy. Results for the Category 1 and Category 2 settings are shown in Fig 3 and Fig 4 of the main text.

**
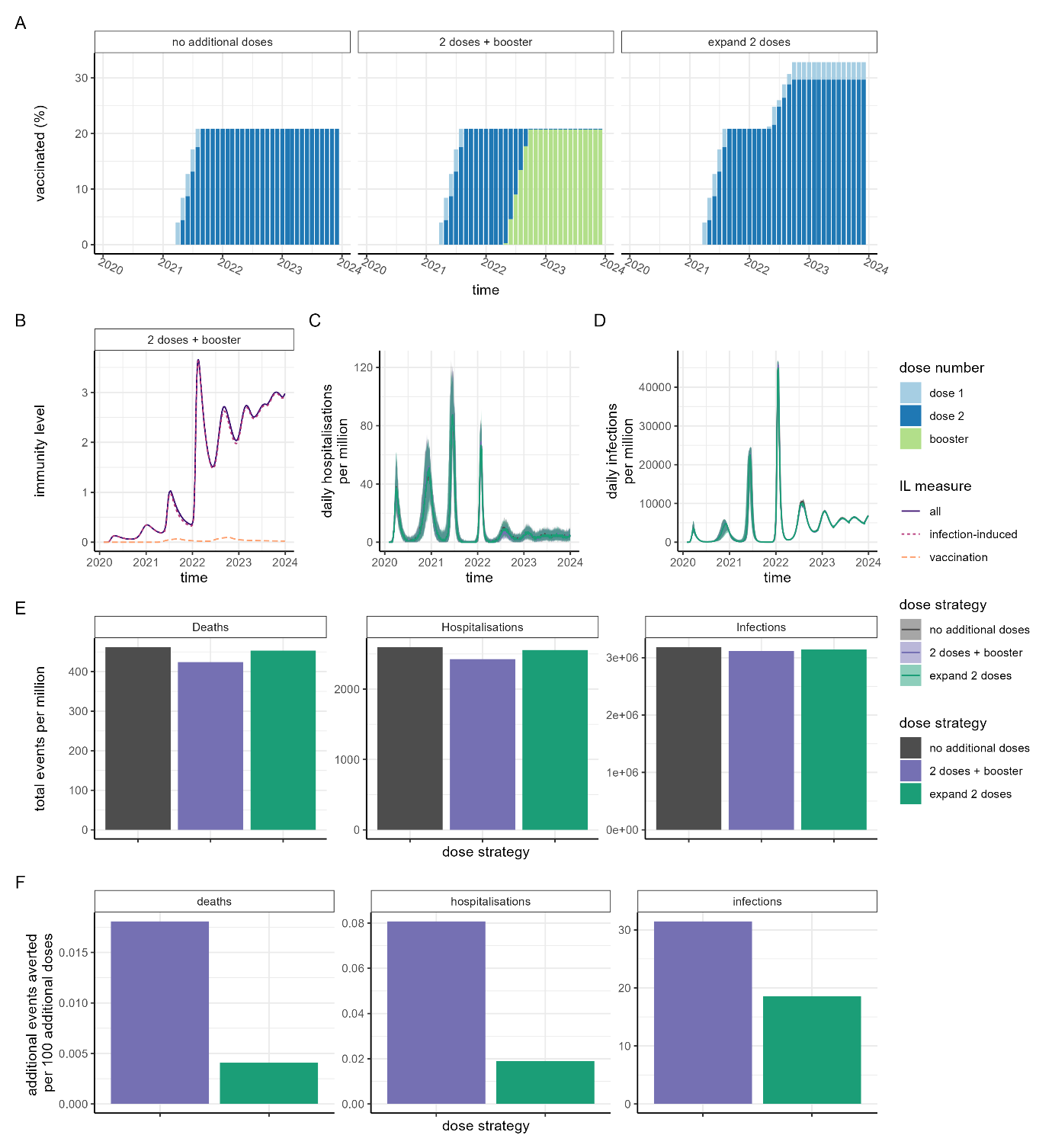
**

**Fig G: Impact of vaccination in a low-middle-income country setting with substantial prior transmission and low vaccine access, where individuals 40+ years are initially targeted.** Three strategies for distributing a limited vaccine supply are shown (assuming AZD1222 is used for all doses). In “expand 2 doses”, no booster doses are administered, and the supply is therefore delivered to a wider proportion of the population. In “2 doses + booster” the same supply is delivered to the 40+ age-group (2 dose primary immunisation and booster dose 12 months post dose 2) and no younger groups receive the primary series. For these two scenarios, the same number of doses are delivered each day, allowing comparison of the impact of different distribution of the same dose supply. We compare with “no additional doses” in which no further doses are rolled out beyond the primary series in 2021. (A) Cumulative proportion of the population receiving dose 1 (light blue), dose 2 (dark blue) and the booster (light green) each month. (B) Mean infection-induced (pink dotted), vaccine-induced (orange dashed), and total (purple solid) immunity level (IL) over time for the “2 doses + booster” dose strategy. (C) Daily hospital admissions; and (D) Daily infections per million population. (E) Total deaths, hospitalisations, and infections per million population from 1 July 2022 to end-2023. (F) Additional events averted per 100 additional doses over the same time period relative to the “No additional doses” strategy. Total events are summarised in Table M.

**
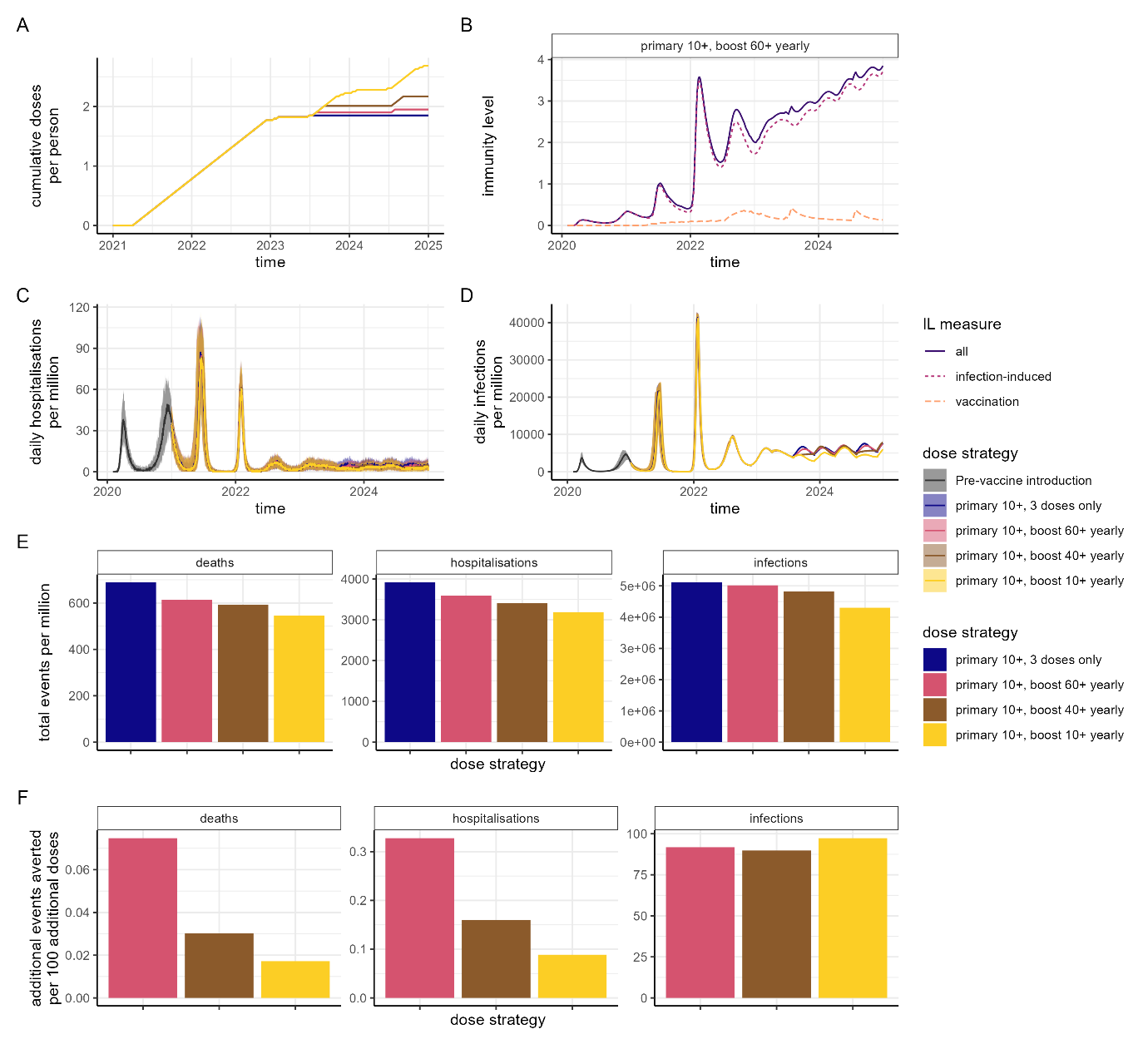
**

**Fig H: Impact of vaccination in a lower-middle-income country setting with substantial prior transmission and moderate vaccine access (Category 2), assuming WHO coverage targets.** We assume AZD1222 is implemented for the first 2 doses, mRNA-1273 for the first booster (dose 3), and a variant-adapted vaccine for subsequent booster doses. (A) Cumulative doses delivered per person over time, for a range of dose delivery strategies. In all strategies, the primary series was delivered to individuals 10 years and older, with scenarios of no additional doses; annual boosters to the 60+ years population; annual boosters to the 40+ years population; or annual boosters to the 10+ years population. (B) Mean infection-induced (pink dotted), vaccine-induced (orange dashed), and total (purple solid) immunity level (IL) over time for the “primary 10+, boost 60+ yearly” dose strategy. (C) Daily hospitalisations and (D) daily infections per million population for the dose strategies, where the trajectory prior to vaccine introduction is shown in dark grey. (E) Total events (deaths, hospitalisations, and infection) per million population between 1 July 2022 and 31 December 2024 for each dose strategy. (F) Additional events averted per 100 additional doses over the same time period relative to the “primary 10+, 3 doses only” dose strategy. Total events are summarised in Table J. WHO: World Health Organization

**
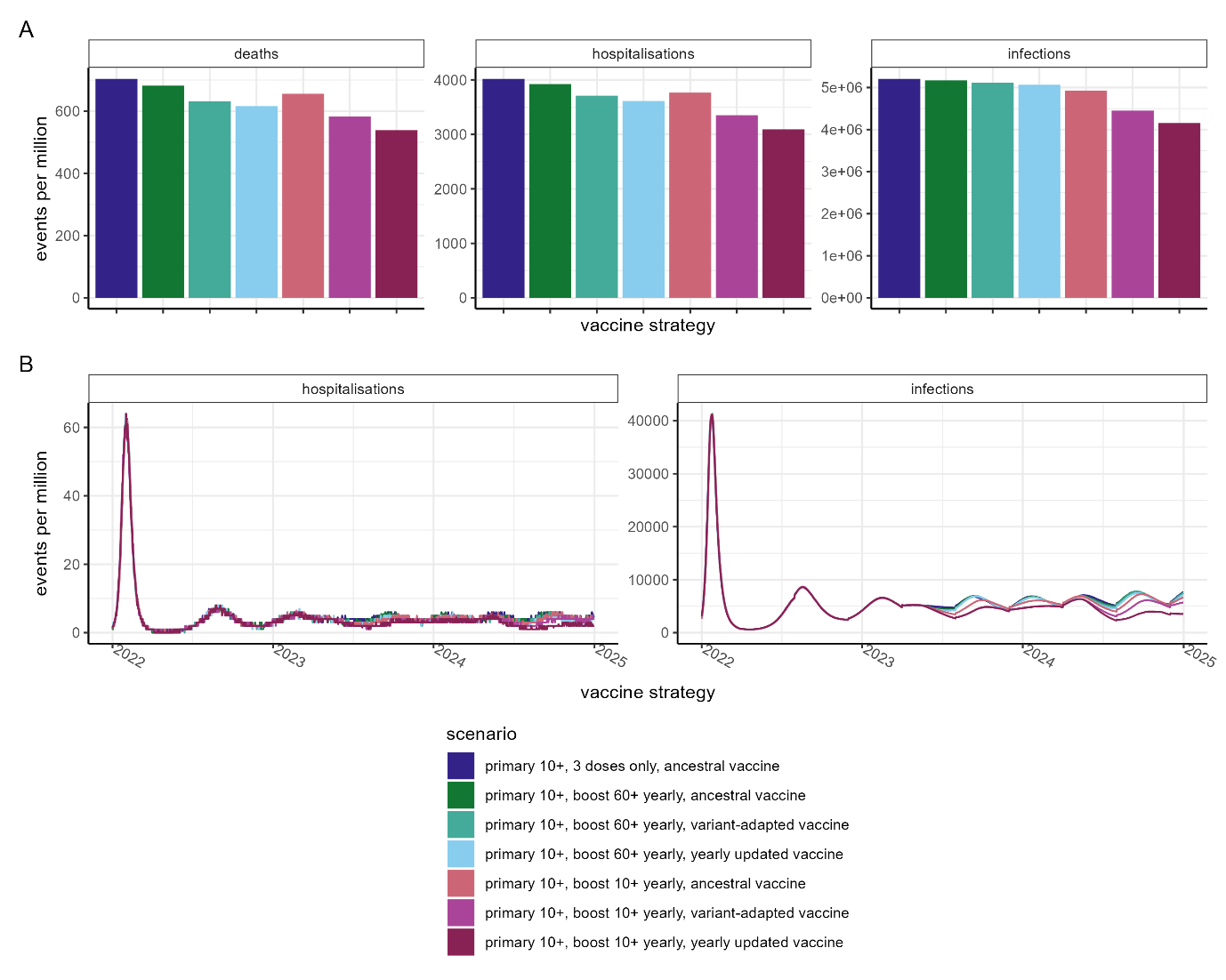
**

**Fig I: Comparison of vaccine impact for different ancestral and variant-adapted vaccine scenarios, for the lower-middle-income country setting with substantial prior transmission (Category 2).** For all vaccine strategies, we assume AZD1222 is implemented for the first 2 doses, and mRNA-1273 for the first booster (dose 3). Following this, either no additional doses are administered, the ancestral mRNA-1273 vaccine is administered for subsequent doses, a variant-adapted vaccine is administered from dose 4, with no additional changes to the vaccine product (“variant-adapted vaccine”), or a variant-adapted vaccine is administered for dose 4, and subsequent doses are continually adapted based on the level of immune escape 12 months beforehand (“yearly updated vaccine”). (A) Summary events (deaths, hospitalisations and infections) per million population for the different vaccine strategies between July 2022 and December 2024, and (B) daily hospitalisations and infections per million population.

**
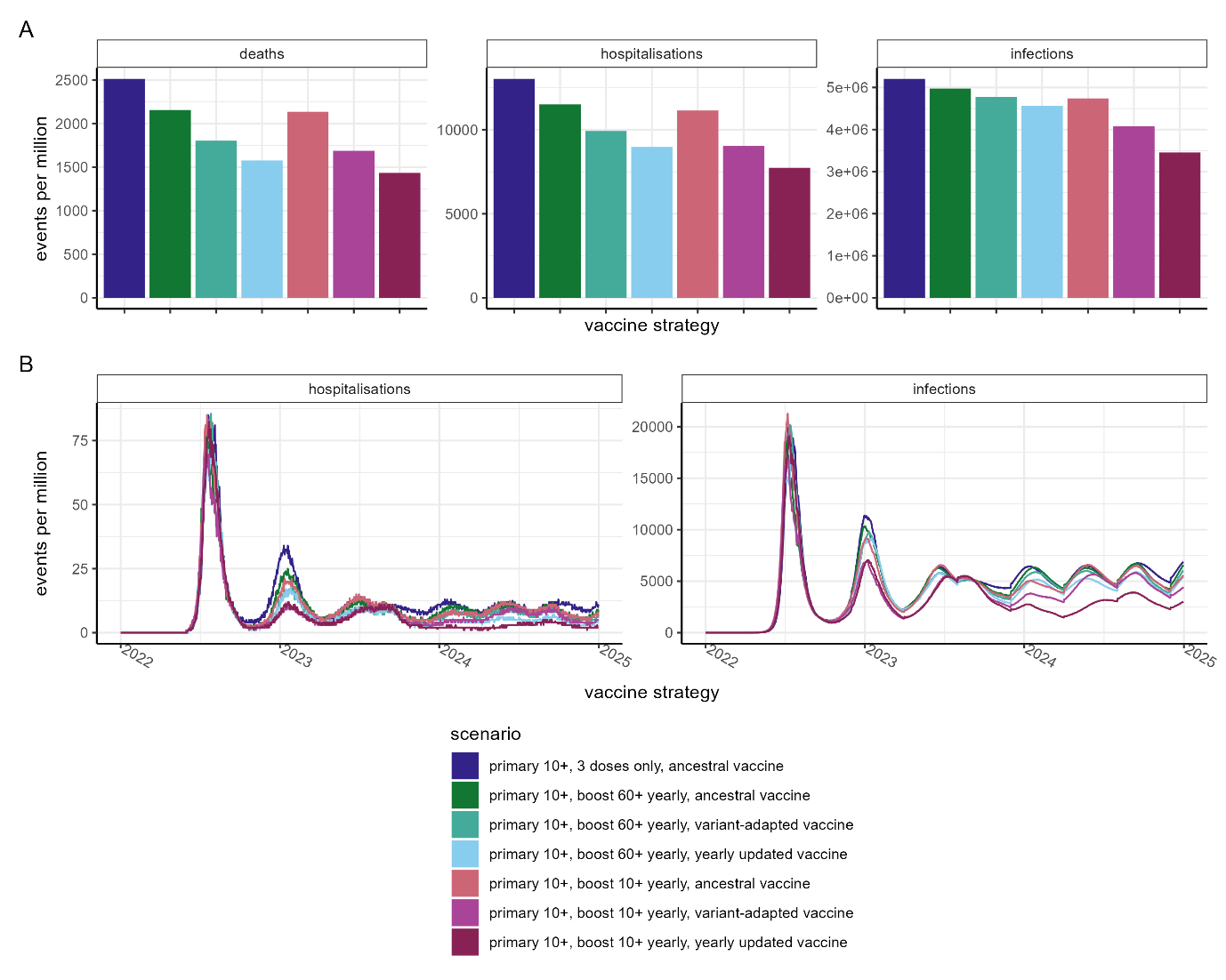
**

**Fig J: Comparison of vaccine impact for different ancestral and variant-adapted vaccine scenarios, for the high-income country setting with minimal prior transmission (Category 3).** For all vaccine strategies, we assume mRNA-1273 is implemented for the first 2 doses and the first booster (dose 3). Following this, either no additional doses are administered, the ancestral vaccine is administered for subsequent doses, a variant-adapted vaccine is administered from dose 4, with no additional changes to the vaccine product (“variant-adapted vaccine”), or a variant-adapted vaccine is administered for dose 4, and subsequent doses are continually adapted based on the level of immune escape 12 months beforehand (“yearly updated vaccine”). (A) Summary events (deaths, hospitalisations and infections) per million population for the different vaccine strategies between July 2022 and December 2024, and (B) daily hospitalisations and infections per million population.

**
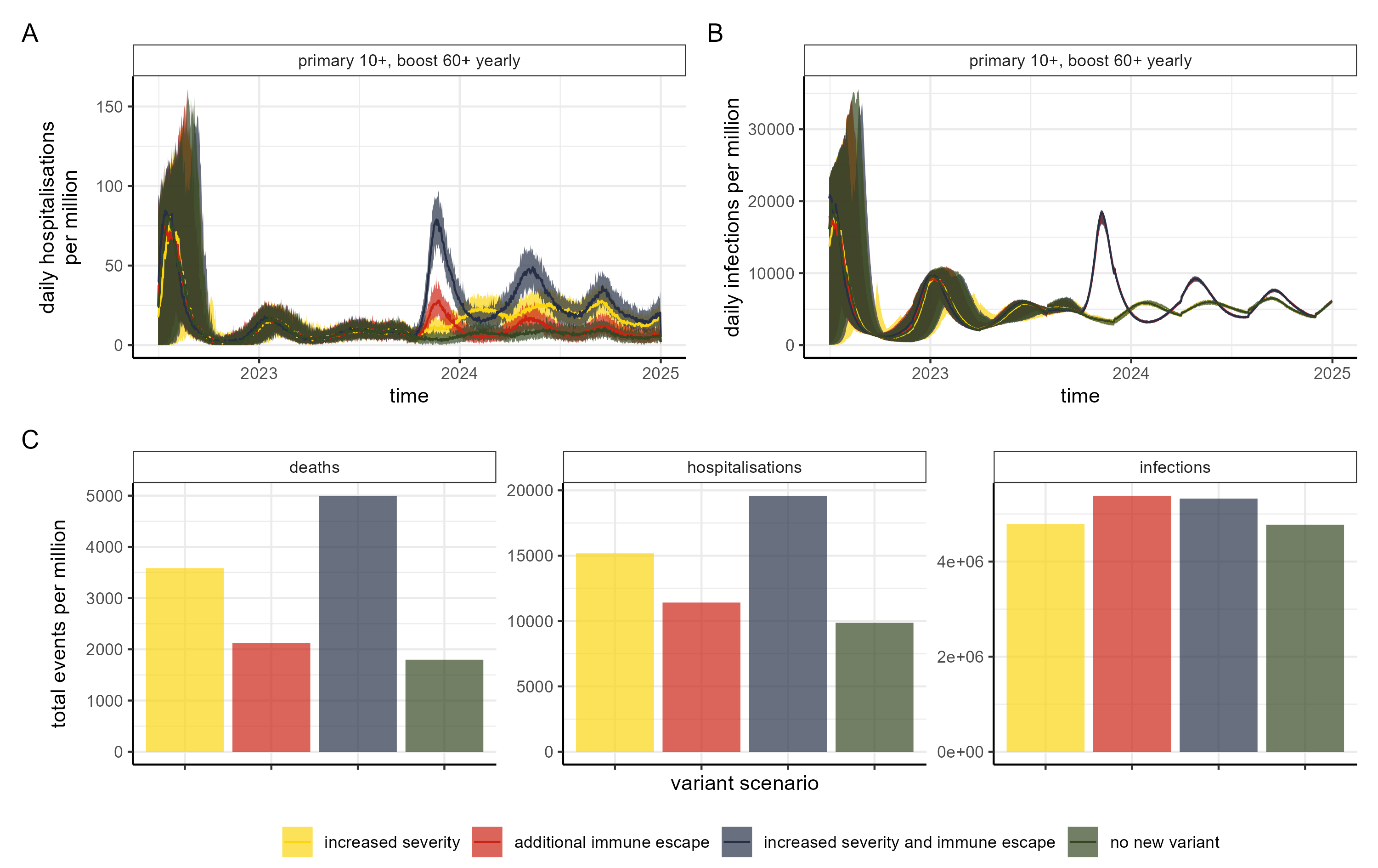
**

**Fig K: Impact of vaccination in future scenarios where an additional variant of concern emerges from 1 October 2022, in a high-income setting with minimal prior transmission and high vaccine access (Category 3).** **We assume a variant-adapted vaccine is implemented from dose 4.** Three variant scenarios are shown: increased severity, where the risk of hospitalisations and severe disease reverts to that of Delta (yellow); additional immune escape, where the variant fold reduction (VFR) increases to 10 (red); and increased severity and immune escape, which assumes both Delta severity and a VFR of 10 (blue). This is compared to the scenario with no new variant (green). (A) Daily hospitalisations, and (B) daily infections per million population; (C) Total events (deaths, hospitalisations and infections) per million population for each variant scenario, between 1 July 2022 and end-2024. Results for the Category 1 and 2 settings are in Fig 6 of the main text.


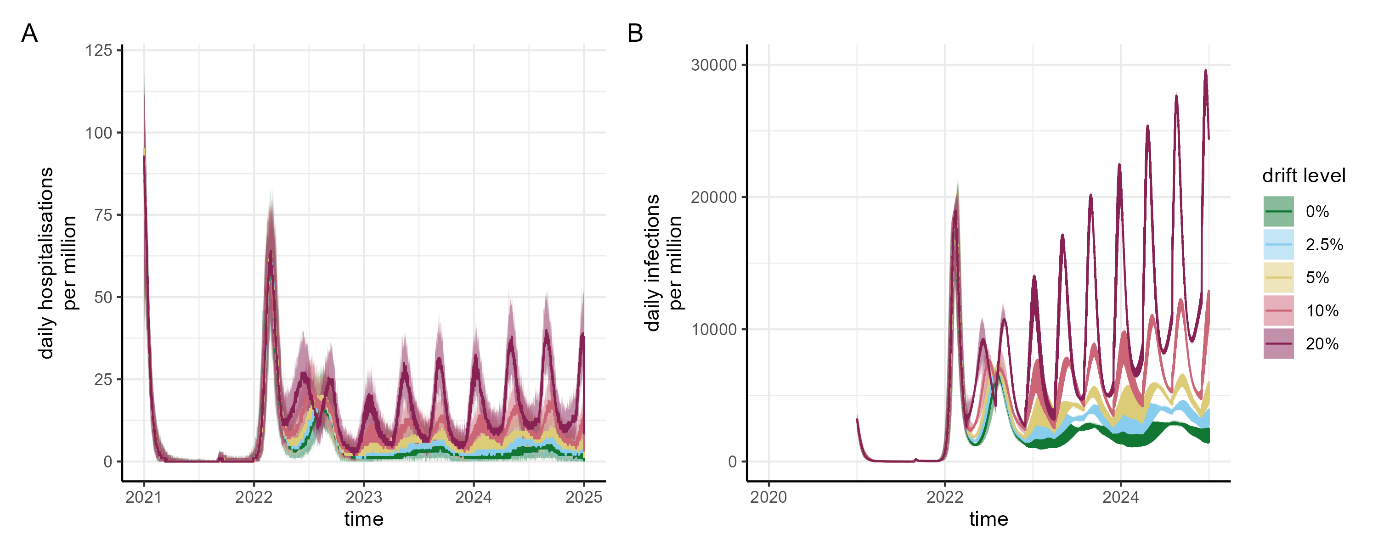


**Fig L: Sensitivity of the model output to the level of “drift” in a high-income setting with substantial prior transmission (Category 1).** This is implemented in the model by increasing both the level of transmission and the VFR relative to Delta every 4 months, to represent both an increase in transmissibility and gradual immune escape. Here we show five levels of transmission and immune escape, from 0% (no drift) to 20%, where 5% (in yellow) is the default level implemented in the main analysis. (A) Daily hospitalisations per million population, and (B) daily infections per million population, assuming the first three doses of the ancestral Moderna vaccine, and annual boosting to the 60+ population with a variant-adapted vaccine.


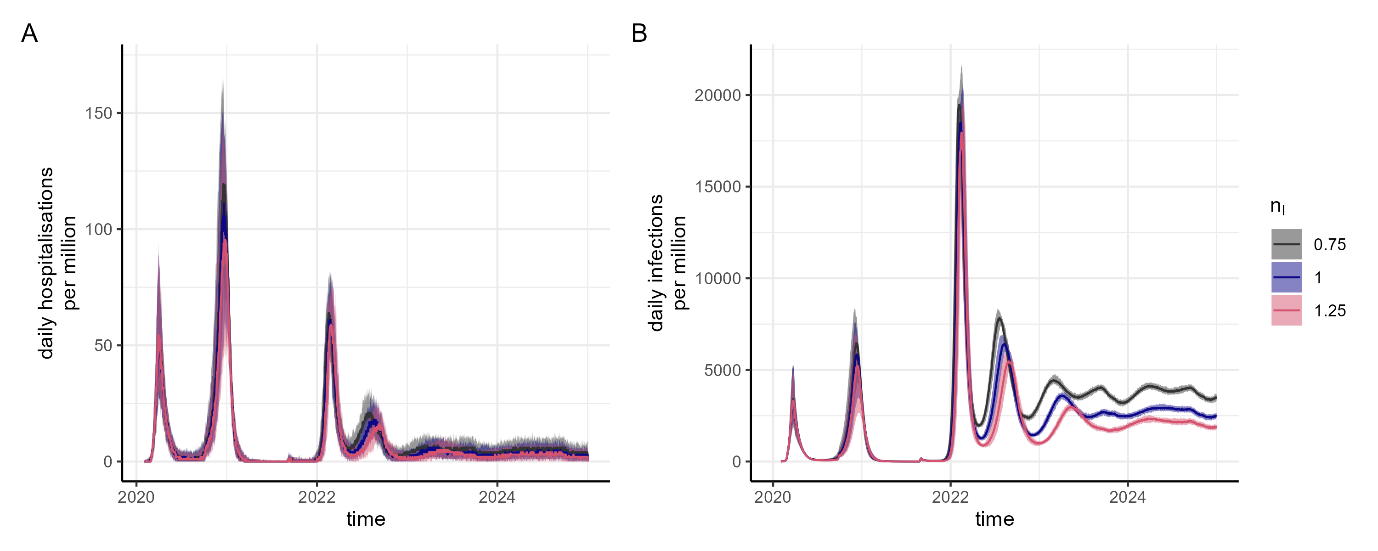


**Fig M: Sensitivity of model results to assumptions regarding the level of protection afforded by infection in a high-income setting with substantial prior transmission (Category 1).** For the main results we assume that infection results in a boost of 1 to immunity levels (purple, 80% protection from re-infection with the same variant over 180 days). Lower boosting (grey = 0.75, 65% protection from re-infection with the same variant over 180 days) results in lower levels of population immunity and higher endemic prevalence; conversely higher infection immunity (pink = 1.25, 89% protection from re-infection with the same variant over 180 days) results in higher population immunity and hence lower endemic prevalence. (A) Daily hospitalisations per million population, and (B) daily infections per million population, assuming the first three doses of the ancestral Moderna vaccine, and annual boosting to the 60+ population with a variant-adapted vaccine.


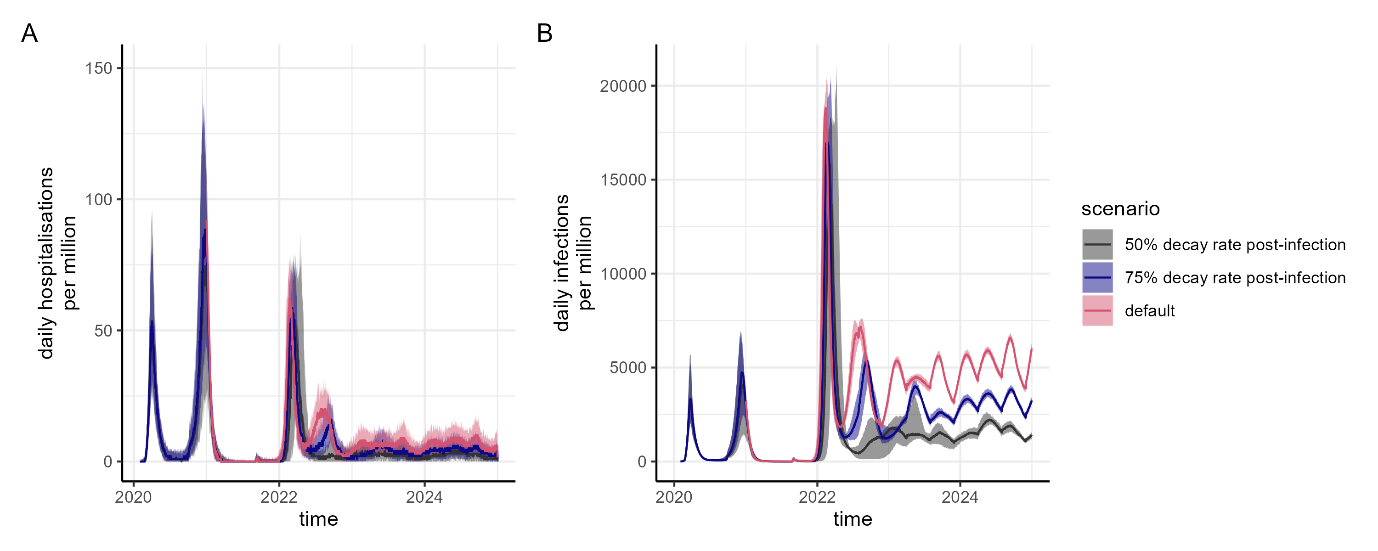


**Fig N: Sensitivity of model results to the decay rate of protection following recovery from SARS-CoV-2 infection in a high-income setting with substantial prior transmission (Category 1).** Each scenario refers to the durability of infection-induced protection relative to that afforded by a vaccine booster dose, where “default” assumes infection-induced protection decays at the same rate as vaccine-induced protection (as is the assumption for the main analysis); and 50% and 75% represent reduced decay, or rather more durable protection. (A) Daily hospitalisations per million population, and (B) daily infections per million population, assuming the first three doses of the ancestral Moderna vaccine, and annual boosting to the 60+ population with a variant-adapted vaccine.

**Table F: Total doses delivered, infections, hospitalisations, and deaths for each of the Category 1 and 3 settings, for a range of vaccine dose strategies and variant scenarios.** **We assume mRNA-1273 is implemented for the first 2 doses and the first booster (dose 3), and a variant-adapted vaccine for subsequent booster doses.** Totals are summarised between 1 July 2022 and 31 December 2024. Unless otherwise specified, we assume no additional variant emergence beyond Omicron and its subtypes. The “new variant worse-case scenario” refers to a scenario where a new variant replaces Omicron over one month, starting on 1 October 2022, with variant fold reduction (VFR) = 10 relative to Delta and severity similar to Delta Values represent the median estimate across 50 model simulations for each scenario. HIC: high-income country setting.

| Vaccination scenario | Doses delivered per million population | Infections per thousand population | Hospitalisations per million population | Deaths per million population |
| --- | --- | --- | --- | --- |
| Setting: HIC with substantial prior transmission and high existing vaccine coverage | | | | |
| 3 doses only | 2014833 | 4727 | 9339 | 1831 |
| Boost 75+ yearly | 2224848 | 4618 | 7862 | 1390 |
| Boost 75+ 6-monthly | 2364858 | 4592 | 7569 | 1300 |
| Boost 60+ yearly | 2690403 | 4326 | 6688 | 1217 |
| Boost 60+ 6-monthly | 3140783 | 4212 | 6144 | 1082 |
| Boost 10+ yearly | 3352017 | 3641 | 5918 | 1122 |
| Boost 60+ yearly, new variant worst-case scenario | 2690403 | 2810 | 8776 | 2236 |
| Boost 10+ yearly, new variant worst-case scenario | 3352017 | 2275 | 7531 | 1984 |
| Setting: HIC with limited prior transmission and high existing vaccine coverage | | | | |
| 3 doses only | 2014833 | 5167 | 13031 | 2536 |
| Boost 75+ yearly | 2224848 | 5095 | 11375 | 2011 |
| Boost 75+ 6-monthly | 2364858 | 4990 | 10858 | 1886 |
| Boost 60+ yearly | 2690403 | 4778 | 9926 | 1804 |
| Boost 60+ 6-monthly | 3140783 | 4708 | 9344 | 1674 |
| Boost 10+ yearly | 3352017 | 4081 | 9040 | 1688 |
| Boost 60+ yearly, new variant worst-case scenario | 2690403 | 5325 | 19583 | 4998 |
| Boost 10+ yearly, new variant worst-case scenario | 3352017 | 4859 | 18656 | 4804 |

**Table G: Total doses delivered, infections, hospitalisations, and deaths for each of the Category 1 and 3 settings, for a range of vaccine dose strategies and variant scenarios. We assume no additional variant emergence beyond Omicron (i.e constant transmission and no additional immune escape, or no “drift”).** We assume mRNA-1273 is implemented for the first 2 doses and the first booster (dose 3), and a variant-adapted vaccine for subsequent booster doses. Totals are summarised between 1 July 2022 and 31 December 2024. Values represent the median estimate across 50 model simulations for each scenario. HIC: high-income country setting.

| Vaccination scenario | Doses delivered per million population | Infections per thousand population | Hospitalisations per million population | Deaths per million population |
| --- | --- | --- | --- | --- |
| Setting: HIC with substantial prior transmission and high existing vaccine coverage | | | | |
| 3 doses only | 2014833 | 2949 | 6338 | 1236 |
| Boost 75+ yearly | 2224848 | 2868 | 5190 | 889 |
| Boost 75+ 6-monthly | 2364858 | 2851 | 4996 | 838 |
| Boost 60+ yearly | 2690403 | 2631 | 4222 | 768 |
| Boost 60+ 6-monthly | 3140783 | 2560 | 3860 | 678 |
| Boost 10+ yearly | 3352017 | 1982 | 3420 | 646 |
| Setting: HIC with limited prior transmission and high existing vaccine coverage | | | | |
| 3 doses only | 2014833 | 3487 | 9874 | 1904 |
| Boost 75+ yearly | 2224848 | 3382 | 8416 | 1490 |
| Boost 75+ 6-monthly | 2364858 | 3363 | 8285 | 1421 |
| Boost 60+ yearly | 2690403 | 3173 | 7366 | 1318 |
| Boost 60+ 6-monthly | 3140783 | 3083 | 6976 | 1208 |
| Boost 10+ yearly | 3352017 | 2448 | 6354 | 1179 |

**Table H: Total doses delivered, infections, hospitalisations, and deaths for the Category 2 setting, for a range of vaccine dose strategies and variant scenarios.** We assume AZD1222 is implemented for the first 2 doses, mRNA-1273 for the first booster (dose 3), and a variant-adapted vaccine for subsequent booster doses (doses 4 and 5). Totals are summarised between 1 July 2022 and 31 December 2024. The “new variant worse-case scenario” refers to a scenario where a new variant replaces Omicron over one month, starting on 1 October 2022, with variant fold reduction (VFR) = 10 relative to Delta and severity similar to Delta. Values represent the median estimate across 50 model simulations for each scenario. LMIC: lower-middle income setting.

| Vaccination scenario | Doses delivered per million population | Infections per thousand population | Hospitalisations per million population | Deaths per million population |
| --- | --- | --- | --- | --- |
| Setting: LMIC with substantial prior transmission and low existing vaccine coverage (default coverage target assumption) | | | | |
| 3 doses only | 1594343 | 5207 | 4046 | 704 |
| Boost 60+ yearly | 1679471 | 5118 | 3709 | 632 |
| Boost 40+ yearly | 1867403 | 4948 | 3564 | 611 |
| Boost 10+ yearly | 2363597 | 4451 | 3351 | 583 |
| Boost 60+ yearly, new variant worst-case scenario | 1679471 | 5754 | 8636 | 2804 |
| Boost 10+ yearly, new variant worst-case scenario | 2363597 | 5256 | 8157 | 2697 |

**Table I: Total doses delivered, infections, hospitalisations, and deaths for the Category 2 setting, for a range of vaccine dose strategies and variant scenarios. We assume no additional variant emergence beyond Omicron (i.e constant transmission and no additional immune escape, or no “drift”).** We assume administration of AZD1222 for the first 2 doses, mRNA-1273 for the first booster dose (dose 3), and a variant-adapted vaccine for all subsequent doses. Totals are summarised between 1 July 2022 and 31 December 2024. Values represent the median estimate across 50 model simulations for each scenario. LMIC: lower-middle income setting.

| Vaccination scenario | Doses delivered per million population | Infections per thousand population | Hospitalisations per million population | Deaths per million population |
| --- | --- | --- | --- | --- |
| Setting: LMIC with substantial prior transmission and low existing vaccine coverage (default coverage target assumption) | | | | |
| 3 doses only | 1594343 | 3216 | 2703 | 474 |
| Boost 60+ yearly | 1679471 | 3132 | 2441 | 414 |
| Boost 40+ yearly | 1867403 | 2955 | 2271 | 393 |
| Boost 10+ yearly | 2363597 | 2413 | 1946 | 346 |

**Table J: Total doses delivered, infections, hospitalisations, and deaths for the Category 2 setting, for a range of vaccine dose strategies and variant scenarios, assuming the WHO coverage target assumption. We assume AZD1222 is implemented for the first 2 doses, mRNA-1273 for the first booster (dose 3), and a variant-adapted vaccine for subsequent booster doses (doses 4 and 5).** Totals are summarised between 1 July 2022 and 31 December 2024. Values represent the median estimate across 50 model simulations for each scenario. LMIC: lower-middle income setting.

| Vaccination scenario | Doses delivered per million population | Infections per thousand population | Hospitalisations per million population | Deaths per million population |
| --- | --- | --- | --- | --- |
| Setting: LMIC with substantial prior transmission and low existing vaccine coverage (WHO coverage target assumption) | | | | |
| 3 doses only | 1850710 | 5111 | 3917 | 688 |
| Boost 60+ yearly | 1949736 | 5020 | 3592 | 614 |
| Boost 40+ yearly | 2168350 | 4826 | 3410 | 592 |
| Boost 10+ yearly | 2683164 | 4301 | 3180 | 546 |

**Table K: Total additional infections, hospitalisations, and deaths averted, and total additional vaccine doses delivered for the Category 1 and 3 settings. We assume no additional variant emergence beyond Omicron (i.e constant transmission and no additional immune escape, or no “drift”).** We assume mRNA-1273 is implemented for the first 2 doses and the first booster (dose 3), and a variant-adapted vaccine for subsequent booster doses. Impact is expressed relative to the scenario where the primary series plus a booster is delivered to the 10+ years population, with no additional doses. Totals are shown for the period from 1 July 2022 to 31 December 2024. Values are the median estimate across 50 model simulations for each scenario. Total modelled events for each scenario are in Table G. HIC: high-income country setting.

| Vaccination scenario | Doses delivered per million population | Infections averted per thousand population | Hospitalisations averted per million population | Deaths averted per million population | Doses to avert one hospitalisation | Doses to avert one death | Cost per hospitalisation averted ($) | | | Cost per death averted ($) | | |
| --- | --- | --- | --- | --- | --- | --- | --- | --- | --- | --- | --- | --- |
| Unit cost per vaccine dose (illustrative) | | | | | | | 2 | 20 | 50 | 2 | 20 | 50 |
| HIC with substantial prior transmission and high existing vaccine coverage | | | | | | | | | | | | |
| Boost 75+ yearly | 210015 | 82 | 1148 | 347 | 183 | 605 | 366 | 3659 | 9147 | 1210 | 12105 | 30262 |
| Boost 75+ 6-monthly | 350025 | 98 | 1342 | 398 | 261 | 879 | 522 | 5216 | 13041 | 1759 | 17589 | 43973 |
| Boost 60+ yearly | 675570 | 318 | 2116 | 468 | 319 | 1444 | 639 | 6385 | 15963 | 2887 | 28871 | 72176 |
| Boost 60+ 6-monthly | 1125950 | 389 | 2478 | 558 | 454 | 2018 | 909 | 9088 | 22719 | 4036 | 40357 | 100892 |
| Boost 10+ yearly | 1337184 | 967 | 2918 | 590 | 458 | 2266 | 917 | 9165 | 22913 | 4533 | 45328 | 113321 |
| HIC with limited prior transmission and high existing vaccine coverage | | | | | | | | | | | | |
| Boost 75+ yearly | 210015 | 105 | 1458 | 414 | 144 | 507 | 288 | 2881 | 7202 | 1015 | 10146 | 25364 |
| Boost 75+ 6-monthly | 350025 | 123 | 1589 | 483 | 220 | 725 | 441 | 4406 | 11014 | 1449 | 14494 | 36234 |
| Boost 60+ yearly | 675570 | 313 | 2508 | 586 | 269 | 1153 | 539 | 5387 | 13468 | 2306 | 23057 | 57642 |
| Boost 60+ 6-monthly | 1125950 | 403 | 2898 | 696 | 389 | 1618 | 777 | 7771 | 19426 | 3235 | 32355 | 80887 |
| Boost 10+ yearly | 1337184 | 1038 | 3520 | 725 | 380 | 1844 | 760 | 7598 | 18994 | 3689 | 36888 | 92220 |

**Table L:** **Total additional infections, hospitalisations, and deaths averted, and total additional vaccine doses delivered for the Category 2 setting. We assume no additional variant emergence beyond Omicron (i.e constant transmission and no additional immune escape, or no “drift”).** We assume administration of AZD1222 for the first 2 doses, mRNA-1273 for the first booster dose (dose 3), and a variant-adapted vaccine for all subsequent doses**.** Impact is expressed relative to the scenario where the primary series plus a booster is delivered to the 10+ years population, with no additional doses. Totals are shown for the period from 1 July 2022 to 31 December 2024. Values are the median estimate across 50 model simulations for each scenario. Total modelled events for each scenario are in Table I. LMIC: lower-middle income setting.

| Vaccination scenario | Doses delivered per million population | Infections averted per thousand population | Hospitalisations averted per million population | Deaths averted per million population | Doses to avert one hospitalisation | Doses to avert one death | Cost per hospitalisation averted ($) | | | Cost per death averted ($) | | |
| --- | --- | --- | --- | --- | --- | --- | --- | --- | --- | --- | --- | --- |
| Unit cost per vaccine dose (illustrative) | | | | | | | 2 | 20 | 50 | 2 | 20 | 50 |
| LMIC with substantial prior transmission and low existing vaccine coverage | | | | | | | | | | | | |
| Boost 60+ yearly | 85128 | 84 | 262 | 60 | 325 | 1419 | 650 | 6498 | 16246 | 2838 | 28376 | 70940 |
| Boost 40+ yearly | 273060 | 260 | 432 | 81 | 632 | 3371 | 1264 | 12642 | 31604 | 6742 | 67422 | 168556 |
| Boost 10+ yearly | 769254 | 803 | 757 | 128 | 1016 | 6010 | 2032 | 20324 | 50809 | 12020 | 120196 | 300490 |

**Table M: Total doses delivered, infections, hospitalisations, and deaths for the Category 2 setting, for a range of vaccine dose strategies, for the scenario where individuals 40+ years are initially targeted. We assume AZD1222 is administered for all doses.** Totals are summarised between 1 July 2022 and 31 December 2023. Three strategies for distributing a limited vaccine supply are shown (assuming AstraZeneca is used for both the primary and booster doses). In “Expand 2 doses”, no booster doses are administered, and the supply is therefore delivered to a wider proportion of the population. In “2 doses + booster” the same supply is delivered to the 40+ age-group (2 dose primary immunisation and booster dose 12 months post dose 2) and no younger groups receive the primary series. For these two scenarios, the same number of doses are delivered each day, allowing comparison of the impact of different distribution of the same dose supply. We compare with “2 doses only to 40+ years” (or no additional doses) in which no further doses are rolled out beyond the primary series in 2021. Values represent the median estimate across 50 model simulations for each scenario. Visualisations for these scenarios are in Fig G. LMIC: lower-middle income setting.

| Vaccination scenario | Doses delivered per million population | Infections per thousand population | Hospitalisations per million population | Deaths per million population |
| --- | --- | --- | --- | --- |
| Setting: LMIC with substantial prior transmission and low existing vaccine coverage, AstraZeneca vaccine | | | | |
| 2 doses only to 40+ years | 417094 | 3182 | 2595 | 462 |
| 2 doses + booster to 40+ years | 624584 | 3116 | 2427 | 424 |
| Expand 2 doses to additional ages | 625582 | 3143 | 2555 | 453 |

**Table N: Total doses delivered, infections, hospitalisations, and deaths for different ancestral, variant-adapted, and yearly updated vaccine scenarios.** For all vaccine strategies, following the primary series and first booster dose with the ancestral vaccine, either no additional doses are administered (“3 doses only”), the ancestral vaccine is administered for subsequent doses, a variant-adapted vaccine is administered from dose 4, with no additional changes to the vaccine product (“variant-adapted vaccine”), or a variant-adapted vaccine is administered for dose 4, and subsequent doses are continually adapted based on the level of immune escape 12 months beforehand (“yearly updated vaccine”). Totals are shown for the period from 1 July 2022 to 31 December 2024. Values are the median estimate across 50 model simulations for each scenario. Results are visualised in Fig 5 of the main text, Fig I, and Fig J. HIC: high-income country setting; LMIC: lower-middle income setting.

| Vaccine type | Vaccination scenario | Doses delivered per million population | Infections per thousand population | Hospitalisations per million population | Deaths per million population |
| --- | --- | --- | --- | --- | --- |
| HIC with substantial prior transmission and high existing vaccine coverage (Category 1) | | | | | |
| Ancestral vaccine | 3 doses only | 2014833 | 4727 | 9334 | 1840 |
|  | Boost 60+ yearly | 2690403 | 4556 | 8083 | 1538 |
|  | Boost 10+ yearly | 3352017 | 4308 | 7794 | 1511 |
| Variant-adapted vaccine | Boost 60+ yearly | 2690403 | 4326 | 6688 | 1217 |
|  | Boost 10+ yearly | 3352017 | 3641 | 5918 | 1122 |
| Yearly updated vaccine | Boost 60+ yearly | 2690403 | 4103 | 5744 | 1009 |
|  | Boost 10+ yearly | 3352017 | 2990 | 4574 | 844 |
| LMIC with substantial prior transmission and low existing vaccine coverage (Category 2) | | | | | |
| Ancestral vaccine | 3 doses only | 1594343 | 5208 | 4020 | 704 |
|  | Boost 60+ yearly | 1679471 | 5171 | 3924 | 682 |
|  | Boost 10+ yearly | 2363597 | 4928 | 3764 | 656 |
| Variant-adapted vaccine | Boost 60+ yearly | 1679471 | 5118 | 3709 | 632 |
|  | Boost 10+ yearly | 2363597 | 4451 | 3351 | 583 |
| Yearly updated vaccine | Boost 60+ yearly | 1679471 | 5067 | 3612 | 616 |
|  | Boost 10+ yearly | 2363597 | 4156 | 3093 | 539 |
| HIC with limited prior transmission and high existing vaccine coverage (Category 3) | | | | | |
| Ancestral vaccine | 3 doses only | 2014833 | 5206 | 13028 | 2514 |
|  | Boost 60+ yearly | 2690403 | 4976 | 11510 | 2156 |
|  | Boost 10+ yearly | 3352017 | 4742 | 11146 | 2136 |
| Variant-adapted vaccine | Boost 60+ yearly | 2690403 | 4778 | 9926 | 1804 |
|  | Boost 10+ yearly | 3352017 | 4081 | 9040 | 1688 |
| Yearly updated vaccine | Boost 60+ yearly | 2690403 | 4565 | 8970 | 1576 |
|  | Boost 10+ yearly | 3352017 | 3456 | 7740 | 1434 |

**Table O: Impact of vaccination in future scenarios where an additional variant of concern emerges from 1 October 2023.** We assume a variant-adapted vaccine is implemented from dose 4 with no additional changes to the vaccine product (i.e. no further updating). Three variant scenarios are shown: increased severity, where the risk of hospitalisations and severe disease reverts to that of Delta; additional immune escape, where the VFR increases to 10; and increased severity and immune escape, which assumes both Delta severity and a VFR of 10. This is compared to the scenario with no new variant. Totals are shown for the period from 1 July 2022 to 31 December 2024. Values are the median estimate across 50 model simulations for each scenario. Results are visualised in Fig 6 of the main text and Fig K. HIC: high-income country setting; LMIC: lower-middle income setting.

| Vaccination scenario | Doses delivered per million population | Infections per thousand population | Hospitalisations per million population | Deaths per million population |
| --- | --- | --- | --- | --- |
| HIC with substantial prior transmission and high existing vaccine coverage (Category 1) | | | | |
| No new variant | 2690403 | 2220 | 3678 | 676 |
| Increased severity | 2690403 | 2441 | 6614 | 1605 |
| Additional immune escape | 2690403 | 2810 | 4692 | 856 |
| Increased severity and immune escape | 2690403 | 2810 | 8776 | 2236 |
| LMIC with substantial prior transmission and low existing vaccine coverage (Category 2) | | | | |
| No new variant | 1679471 | 5118 | 3743 | 647 |
| Increased severity | 1679471 | 5118 | 6902 | 2156 |
| Additional immune escape | 1679471 | 5756 | 4384 | 764 |
| Increased severity and immune escape | 1679471 | 5754 | 8636 | 2804 |
| HIC with limited prior transmission and high existing vaccine coverage (Category 3) | | | | |
| No new variant | 2690403 | 4776 | 9872 | 1796 |
| Increased severity | 2690403 | 4787 | 15180 | 3586 |
| Additional immune escape | 2690403 | 5384 | 11414 | 2125 |
| Increased severity and immune escape | 2690403 | 5325 | 19583 | 4998 |

# S3. Supplementary references

1. Khoury DS, Cromer D, Reynaldi A, Schlub TE, Wheatley AK, Juno JA, et al. Neutralizing antibody levels are highly predictive of immune protection from symptomatic SARS-CoV-2 infection. Nat Med. 2021;27: 1205–1211. doi:10.1038/s41591-021-01377-8

2. Khoury DS, Docken SS, Subbarao K, Kent SJ, Davenport MP, Cromer D. Predicting the efficacy of variant-modified COVID-19 vaccine boosters. Nat Med. 2023. doi:10.1038/s41591-023-02228-4

3. Hogan AB, Doohan P, Wu SL, Mesa DO, Toor J, Watson OJ, et al. Estimating long-term vaccine effectiveness against SARS-CoV-2 variants: a model-based approach. Nat Commun. 2023;14. doi:10.1038/s41467-023-39736-3

4. Walker PGT, Whittaker C, Watson OJ, Baguelin M, Winskill P, Hamlet A, et al. The impact of COVID-19 and strategies for mitigation and suppression in low- and middle-income countries. Science. 2020;369: 413–422. doi:10.1126/SCIENCE.ABC0035

5. Hogan AB, Winskill P, Watson OJ, Walker PGT, Whittaker C, Baguelin M, et al. Within-country age-based prioritisation, global allocation, and public health impact of a vaccine against SARS-CoV-2: A mathematical modelling analysis. Vaccine. 2021;39: 2995–3006. doi:10.1016/J.VACCINE.2021.04.002

6. Regev-Yochay G, Gonen T, Gilboa M, Mandelboim M, Indenbaum V, Amit S, et al. Efficacy of a Fourth Dose of Covid-19 mRNA Vaccine against Omicron. New England Journal of Medicine. 2022;386: 1377–1380. doi:10.1056/NEJMc2202542

7. Nyberg T, Ferguson NM, Nash SG, Webster HH, Flaxman S, Andrews N, et al. Comparative analysis of the risks of hospitalisation and death associated with SARS-CoV-2 omicron (B.1.1.529) and delta (B.1.617.2) variants in England: a cohort study. The Lancet. 2022;399: 1303–1312. doi:10.1016/s0140-6736(22)00462-7

8. Watson OJ, Barnsley G, Toor J, Hogan AB, Winskill P, Ghani AC. Global impact of the first year of COVID-19 vaccination: a mathematical modelling study. Lancet Infect Dis. 2022;22: 1293–1302. doi:10.1016/S1473-3099(22)00320-6

9. World Health Organization. WHO Coronavirus (COVID-19) Dashboard. 2023 [cited 20 Jun 2023]. Available: https://covid19.who.int/table

10. World Health Organization. Strategy to Achieve Global Covid-19 Vaccination by mid-2022. 2021. Available: https://www.who.int/publications/m/item/strategy-to-achieve-global-covid-19-vaccination-by-mid-2022
